# Post-onset intermittent fasting attenuates neuroinflammation and demyelination via a TRIB3–PERK–autophagy axis in an EAE model of multiple sclerosis

**DOI:** 10.1186/s12974-025-03640-y

**Published:** 2025-11-27

**Authors:** Weitai He, Xueli Liu, Di Wang, Ye Gong, Tingting Cui, Xin Zhang, Pei Li, Xiaoli Ding, Luting Yang, Qian Zhang, Yang Yang, Xiaochang Xue, Lin Shi, Yaling Zhang, Yaping Yan

**Affiliations:** 1 Key Laboratory of the Ministry of Education for Medicinal Resources and Natural Pharmaceutical Chemistry, National Engineering Laboratory for Resource Development of Endangered Crude Drugs in Northwest of China, College of Life Sciences, Shaanxi Normal University, Xi’an, 710062 China; 2 Institute of Sports Biology, Shaanxi Normal University, Xi’an, 710062 China; 3 School of Food Engineering and Nutritional Science, Shaanxi Normal University, Xi’an, 710062 China

**Keywords:** Experimental autoimmune encephalomyelitis, Intermittent fasting, TRIB3, PERK signaling pathway, Autophagy

## Abstract

Prior studies have demonstrated therapeutic benefits of intermittent fasting (IF) in experimental autoimmune encephalomyelitis (EAE), yet they have predominantly examined prophylactic protocols or implemented short-term post-induction interventions, leaving the therapeutic window undefined and the underlying mechanisms unelucidated. Here, we systematically evaluate intermittent fasting (IF) initiated at a clinically critical juncture of 10 days post-induction (EAE_postIF), which coincides with early symptom onset, demonstrating significant attenuation of disease progression, reduced neuroinflammation, and preserved myelin integrity. Mechanistically, EAE_postIF activates a TRIB3–PERK–autophagy axis in the spinal cord, evidenced by increased ATF4, CHOP, and TRIB3 expression and suppression of mTOR signaling. In TRIB3-deficient mice, the beneficial effects of IF are partially attenuated, with clinical and histological improvements reduced relative to wild-type controls yet remaining superior to untreated cohorts. These findings establish a well-defined therapeutic window for IF intervention in neuroinflammation and identify TRIB3–PERK–autophagy signaling as a critical mediator, supporting IF as a viable metabolic strategy to complement existing MS therapies.

**Supplementary Information:**

The online version contains supplementary material available at 10.1186/s12974-025-03640-y.

## Introduction

Multiple sclerosis (MS) is a chronic immune-mediated demyelinating disease of the central nervous system (CNS) characterized by autoreactive lymphocytes attacking the myelin sheath [1–4], leading to neuroinflammation, axonal injury, and progressive neurological disability [5, 6]. The etiopathogenesis of MS involves a complex interplay of genetic susceptibility and environmental triggers that drive aberrant immune responses against CNS components [6]. Despite advances in immunomodulatory therapies, MS remains incurable, and patients often accumulate disability over time, especially in progressive phases where neurodegeneration continues independent of overt relapses [6–8]. This therapeutic gap has spurred interest in complementary strategies targeting neuroprotective and regenerative pathways beyond conventional immune suppression.

Among environmental factors, diet has emerged as a modifiable element that can influence MS outcomes. Epidemiological studies suggest that healthy dietary patterns (e.g. Mediterranean diet) correlate with reduced MS severity [6]. In particular, intermittent fasting (IF), defined by periodic intervals of caloric abstinence, has gained significant interest due to its profound effects on metabolism and immunity. In addition to fasting, other dietary restriction strategies, such as continuous caloric restriction diets, have also been shown to alleviate EAE severity [9, 10]. Whether the protective effects of these regimens arise from the fasting pattern itself or from an overall reduction in caloric intake remains an open question in the field. Preclinical studies in experimental autoimmune encephalomyelitis (EAE), the principal MS animal model, demonstrate that IF can ameliorate disease: alternate-day fasting or fasting-mimicking diets markedly reduce clinical severity, CNS inflammatory infiltrates, and demyelination [11, 12]. These benefits are accompanied by shifts in the immune milieu, including increased regulatory T-cell responses and enhanced remyelination, as well as alterations in the gut microbiota that favor an anti-inflammatory state [12]. Early clinical evidence is also promising, with small trials in MS patients demonstrating that IF regimens are feasible and can enhance patient-reported outcomes (PROs) alongside beneficial immunological marker profiles [13, 14]. A recent systematic review of IF in MS concluded that this dietary approach is a safe and promising adjunct to manage symptoms and possibly slow disease progression, though larger trials are needed to confirm efficacy [6, 15, 16]. Notably, to date most IF interventions in animal models have been initiated prophylactically (weeks before or at the time of disease induction), leaving it uncertain whether introducing IF after disease onset can still meaningfully influence the neuroinflammatory course. This unanswered question is critical for clinical translation, since patients are diagnosed after disease initiation.

At a mechanistic level, nutritional challenges such as IF are thought to engage cellular stress adaptation pathways that confer neuroprotection. One key pathway is the endoplasmic reticulum (ER) stress response, particularly the PKR-like ER kinase (PERK) branch of the unfolded protein response. PERK activation attenuates global protein synthesis and induces a cadre of cytoprotective genes to restore proteostasis during stress [17]. In the context of MS, ER stress is a significant factor, as oligodendrocytes in active lesions exhibit an accumulation of misfolded proteins due to inflammatory cytokines and oxidative stress. Enhancing PERK-mediated integrated stress response signaling promotes oligodendrocyte survival and functional maintenance during autoimmune attack [17, 18]. For example, enhanced PERK signaling in oligodendrocytes protected these cells from IFN-γ–mediated damage and promoted remyelination in EAE [17]. Pharmacological upregulation of the integrated stress response (e.g. with guanabenz, which prolongs PERK pathway signaling) likewise preserved oligodendrocyte viability and alleviated clinical EAE severity, pointing to the therapeutic potential of targeting this pathway [19–21]. These findings position the PERK arm of the stress response as a tantalizing target whereby a metabolic intervention might counteract the neural injury in MS.

Another major cytoprotective pathway influenced by metabolic interventions is autophagy, an intracellular degradation and recycling process [22, 23]. Autophagy helps maintain cellular homeostasis by clearing damaged organelles and protein aggregates, thereby reducing oxidative stress and curbing hyperactive inflammation [5, 24]. In MS, autophagy exhibits a dual role: basal or adaptive autophagy is largely beneficial, limiting demyelination and promoting remyelination through enhanced oligodendrocyte function; conversely, excessive or dysregulated autophagy may contribute to tissue damage, for instance, via hyperactivation of microglia and lymphocytes [5]. The net effect of autophagy thus depends on its calibration, with studies noting that augmenting autophagic flux can dampen inflammatory cytokine release and protect neurons, but over-activated autophagy correlates with worse pathology [5]. Notably, nutrient deprivation is one of the most potent inducers of autophagy: even short-term fasting triggers robust autophagic responses in neurons and other cell types [25, 26]. Repetitive fasting cycles (such as IF) can thereby activate autophagy-mediated clearance mechanisms, which is postulated to help resolve intracellular stress and immune-mediated damage in neuroinflammatory conditions. Indeed, IF and caloric restriction regimens have been shown to induce “adaptive” autophagy that promotes cellular survival and longevity [11, 25]. This crosstalk between the PERK pathway and autophagy is of particular interest, as PERK activation (through its downstream effector ATF4) can upregulate autophagy genes and inhibitors of mTOR, aligning these two stress-response mechanisms [27, 28]. We hypothesize that IF exerts neuroprotective effects in part by synchronously engaging the PERK–autophagy axis, creating a cellular state more resilient to the inflammatory and metabolic perturbations of EAE/MS.

In this study, we address a vital gap by testing whether an IF regimen, initiated after disease onset, can still ameliorate EAE progression and through what mechanisms. We focus on the PERK–autophagy signaling axis as a novel interface between dietary intervention and MS pathophysiology. Our approach leverages alternate-day fasting beginning in early symptomatic EAE, combined with molecular analyses and a genetic knockout model (Trib3-deficient mice) to dissect the contributions of the ER stress and autophagy pathways. By clearly defining the impact of EAE_postIF on neuroinflammation and demyelination, and identifying the PERK–autophagy axis as a key mediator, this work establishes a proof-of-concept for a novel therapeutic avenue in MS. Our findings highlight how a timed metabolic intervention can engage endogenous protective programs in the CNS, offering a novel therapeutic strategy that addresses an unmet need in treating established MS.

## Materials and methods

### Mice and EAE induction

C57BL/6J mice were purchased from the Laboratory Animal Center of Shaanxi Normal University (Xi’an, China), and C57BL/6 N mice were obtained from Beijing Huafukang Biotechnology Co., Ltd. (Beijing, China). Trib3 heterozygous knockout (Trib3^⁺/⁻^) mice were purchased from Cyagen Biosciences (Guangzhou, China) (strain number KOCMP-228775-Trib3-B6N-VA, NCBI ID: 228775, catalog # S-KO-06197). Homozygous Trib3^⁻/⁻^ mice and corresponding wild-type (WT) littermates were obtained by breeding Trib3^⁺/⁻^ heterozygotes with C57BL/6 N WT mice, followed by intercrossing of the heterozygous offspring. All mice were housed under specific-pathogen-free (SPF) conditions (24 ± 2 °C; 12 h light/dark cycle) and were group-housed, five per cage. Food was provided according to the experimental design (ad libitum, time-restricted, or calorie-restricted feeding), and water was available ad libitum. All procedures were approved by the Animal Research and Ethics Committee of Shaanxi Normal University and complied with institutional and national guidelines for animal welfare. Mice were acclimated for at least one week before experimentation and randomly assigned to experimental groups (n values provided in figure legends). All animal experiments were designed and reported in accordance with the ARRIVE 2.0 guidelines. Female mice aged 8 weeks were used for EAE induction to ensure consistency across experimental groups, as female C57BL/6 mice are more susceptible and display a more reproducible EAE phenotype than males [29, 30]. Active experimental autoimmune encephalomyelitis (EAE) was induced in C57BL/6 mice by immunization with the MOG_35 − 55_ peptide emulsified in complete Freund’s adjuvant (CFA) containing Mycobacterium tuberculosis H37Ra, followed by pertussis toxin (PTX) injections. Briefly, MOG_35–55_ powder was dissolved in sterile water to prepare a 50 mg/mL stock, which was then diluted in PBS to 2 mg/mL working solution. Equal volumes of MOG solution and CFA (containing ~ 1 mg/mL MT H37Ra) were emulsified by mechanical homogenization (e.g. 6 × 30 s bead-beating) until a stable emulsion formed (verified by drop test in water). Each mouse received a total of 200 µg MOG_35–55_ (100 µL per injection site) subcutaneously at 2 flank/back sites. Immediately after immunization (day 0) and again 48 h later, 200 ng of pertussis toxin (in 200 µL PBS, IP) were administered to further enhance blood–brain barrier permeability and disease induction. This active immunization protocol (MOG/CFA + PTX) is a standard C57BL/6 EAE model.

### Clinical scoring

Mice were examined daily from day 7 post-immunization onward for neurological deficits by two blinded observers. A 0–5 clinical scoring scale was used (no symptoms to moribund) with 0.5-point increments for intermediate signs. Criteria were: 0 = normal; 0.5 = tail tip atony; 1 = limp tail; 1.5 = partial hind limb weakness; 2 = hind limb paresis; 2.5 = severe paresis (one limb paralyzed, other weak); 3 = bilateral hind limb paralysis; 3.5 = hind limb paralysis plus partial forelimb weakness; 4 = complete paralysis (hind and forelimbs, mouse cannot right itself); 5 = moribund or dead. These scores reflect ascending paralysis in EAE. Body weight was recorded concurrently. Cumulative and average clinical scores were calculated for each group. Mice reaching a score of 4 or above were monitored closely and euthanized if humane endpoints were met, which were defined as moribund state or inability to access food and water (score = 5). In our cohort, no mice reached this endpoint; therefore, all animals, including those that transiently scored 4, were retained in the clinical scoring dataset and were available for downstream analyses. The scoring and endpoint criteria followed established institutional guidelines for EAE monitoring and humane care [31, 32].

### Intermittent fasting regimen

Intermittent fasting (IF) was implemented as an alternate-day feeding schedule. In this regimen, mice underwent 24 h of food deprivation (“fasting day”) followed by 24 h of ad libitum (AL) feeding (“feeding day”). Water was available at all times. Four feeding groups were established: (1) EAE_AL: EAE induction, fed ad libitum throughout; (2) EAE_postIF: Immunized on day 0, ad libitum feeding until day 10, then IF every other day from day 10 until endpoint; (3) EAE_IF: Immunized on day 0, IF every other day from day − 14 until endpoint; (4) EAE_preIF: Immunized on day 0, IF every other day from day − 14 to day 10, followed by ad libitum feeding thereafter.

On “feeding days,” food pellets were placed in the cage at 17:00; on “fasting days,” pellets were removed at 17:00. Food intake and body weight were monitored daily. Alternate-day fasting has been shown to improve metabolic and inflammatory parameters in rodents.

### Post-onset time-restricted feeding (EAE_postTR) and mild caloric restriction (EAE_postCR) regimen

EAE_postTR: Mice were immunized on day 0 and maintained on ad libitum feeding until day 10. From day 10 until the experimental endpoint, they were subjected to a time-restricted feeding (TR) regimen consisting of a 16 h fasting and 8 h feeding cycle. The feeding window was set from 8:00 p.m. to 4:00 a.m., corresponding to the dark (active) phase of the circadian cycle. During the feeding period, mice had free access to standard chow, and all mice had ad libitum access to water throughout the experiment.

EAE_postCR: Mice were immunized on day 0 and maintained on ad libitum feeding until day 10. From day 10 until the endpoint, mice received a moderate caloric restriction (CR) regimen, in which the amount of chow provided was adjusted to 70% of the average daily food intake of ad libitum-fed EAE_AL mice recorded over the corresponding period. The baseline consumption data were derived from previously established EAE_AL cohorts with continuous food intake monitoring from day 0 to the endpoint, allowing dynamic adjustment of food quantity. All mice had ad libitum access to water throughout the experiment. Mice were group-housed (five per cage) under controlled temperature and light/dark conditions. Daily food and water intake were recorded per cage, and the mean consumption per mouse was calculated by dividing the total cage intake by the number of mice.

### Tissue harvest and processing

At the experiment endpoint, mice were deeply anesthetized (100 µL 1% pentobarbital IP) and transcardially perfused with cold PBS to clear blood, followed by 4% paraformaldehyde (PFA) in PBS. Spinal cords were dissected. For histology/immunostaining, tissues were post-fixed in 4% PFA overnight at 4 °C, then either cryoprotected in sucrose and embedded in OCT for frozen sectioning or processed for paraffin embedding. For molecular analyses (flow cytometry or Western blot), fresh tissues (spinal cord and spleen) were collected on ice without fixation. Spleens were mashed through a 70 μm strainer to obtain single-cell suspensions. Spinal cords were collected from perfused mice and processed immediately for mononuclear cell isolation. Tissues were minced into approximately 1 mm fragments and centrifuged at 400 × g for 3 min to remove debris. The supernatant was discarded, and enzymatic dissociation was performed using the Neural Tissue Dissociation Kit (P) (Miltenyi Biotec, Cat# 130-107-677) according to the manufacturer’s protocol. Briefly, for each spinal cord sample, 50 µL of Enzyme P, 10 µL of Enzyme A, 1900 µL of Buffer Z, and 20 µL of Buffer A were added and incubated at 37 °C, 90 rpm for 30 min with gentle trituration every 10 min. After digestion, 35 mL of PBS was added to stop the reaction, and the suspension was filtered through a 70 μm strainer and centrifuged at 400 × g for 5 min. The resulting pellet was resuspended in 5 mL of 70% Percoll (Cytiva, Cat# 17089109), overlaid with 5 mL of 30% Percoll, and centrifuged at 800 × g for 20 min (acceleration = 9, deceleration = 1) without brake. Mononuclear cells were collected from the interphase between the 30% and 70% layers, washed with PBS, and resuspended for downstream analyses. Red blood cells in splenocyte preparations were lysed with RBC lysis buffer (ACK). Cell counts were determined by hemocytometer, and viability by trypan blue. Typically, 1–2 × 10^5^ cells per sample were used for flow cytometry.

### Histological staining

Spinal cord segments were fixed, then cut into 7 μm sections. For inflammation assessment, sections were stained with Hematoxylin and eosin (H&E) using a standard kit (Servicebio, Cat# G1005). For myelin visualization, adjacent sections were stained with Luxol Fast Blue (LFB) (Solarbio, Cat# G1460) followed by cresyl violet or eosin counterstain. Demyelination was evaluated by the degree of LFB loss and vacuolation. Inflammatory infiltrates and lesion scores were graded by a blinded observer on a 0–3 scale according to published criteria. For H&E staining, inflammation was scored as follows: 0 = no inflammatory cells; 1 = few scattered cells; 2 = perivascular infiltrates; 3 = dense perivascular cuffing extending into the parenchyma. For LFB staining, demyelination was graded as 0 = no demyelination; 1 = focal demyelination; 2 = multiple demyelinated areas; 3 = extensive or confluent demyelination [33, 34].

### Immunofluorescence staining

For cellular localization of inflammation, cryosections (7 μm) of PFA-fixed spinal cord were prepared. Sections were air-dried for 30 min, rinsed in PBS (4 × 5 min washes), and permeabilized in ice-cold acetone for 10 min. After drying, sections were encircled with a hydrophobic barrier (PAP pen), and incubated in blocking buffer (3% BSA, 0.2% Triton X-100 in PBS) for 30 min at room temperature. Primary antibodies (anti-IBA1, anti-GFAP, anti-MBP) diluted in blocking buffer were applied to sections and incubated overnight at 4 °C. After four washes (PBS, 5 min each), fluorescently labeled secondary antibodies (Alexa Fluor 488 or 594 conjugates) were applied (1 h at room temperature, dark). Sections were then washed (4 × 5 min in PBS), stained with DAPI (300 nM in PBS, 5 min), rinsed, and mounted with antifade medium. Stained sections were imaged by fluorescence microscopy. Cell counts and area quantification (percent IBA1⁺ or GFAP⁺ area) were performed using ImageJ (NIH). For each mouse, one representative spinal cord section at a comparable lumbar level was analyzed. In each section, five corresponding, non-overlapping regions of interest (ROIs) encompassing both gray and white matter were selected at identical anatomical locations across animals. The mean value of the five ROIs was used as the representative value for each animal (n = animals). All images were acquired and analyzed under identical parameters across groups. For MBP immunostaining, myelin integrity was semiquantitatively evaluated using a 0–3 pathological scoring system based on established EAE histopathological criteria [35], where 0 = normal myelination, 1 = focal myelin loss, 2 = multiple demyelinated areas, and 3 = extensive or confluent demyelination. Detailed information on primary antibodies for immunofluorescence is provided in Supplementary Table S1.

### Flow cytometry

Mononuclear cells isolated from spinal cord and spleen were analyzed by flow cytometry to quantify T cell subsets. For surface staining, cells were resuspended in FACS buffer (PBS + 2% FBS) and incubated with fluorescently conjugated anti-CD4 (PerCP) antibody or isotype controls for 30 min at room temperature in the dark. For intracellular cytokine staining, cells were first stimulated with PMA (50 ng/mL) and ionomycin (500 ng/mL) in the presence of brefeldin A (GolgiStop) for 4 h at 37 °C. After stimulation, cells were washed (FACS buffer, 3×), surface-stained for CD4 as above, then fixed and permeabilized using a commercial kit (e.g. BD Cytofix/Cytoperm) following manufacturer instructions. Cells were then stained with anti-IL-17 A (APC) and anti-IFN-γ (PE) antibodies for 30 min (dark), washed (Perm/Wash buffer 2×), and resuspended in PBS for acquisition. In parallel, a fraction of cells (unstimulated) was fixed/permeabilized and stained for intracellular FoxP3 (PE) to identify regulatory T cells, following the same wash/incubation steps. Samples were acquired on a BD FACSAria flow cytometer. Compensation was set using single-stained controls. Data were analyzed with FlowJo v10 (BD), gating on lymphocytes (FSC/SSC), single cells, then CD4^+^ T cells, with further gating for IL-17 A^+^ (Th17), IFN-γ^+^(Th1), and FoxP3^+^ (Tregs) subsets. The percentages of cytokine-positive cells among CD4^+^ T cells were reported. This protocol follows established methods for CNS and splenocyte flow analysis in EAE. The antibodies used for flow cytometry are listed in Supplementary Table S2. Although we did not use a dedicated live/dead viability dye, viable cells were selected based on FSC/SSC characteristics and singlet discrimination. This approach is documented in the literature for CNS-derived single-cell suspensions [36, 37].

### Western blot analysis

Spinal cord tissues were lysed in RIPA buffer containing protease and phosphatase inhibitors (Roche) on ice. Samples were sonicated (1 min) and centrifuged (12,000 ×g, 10 min, 4 °C) to pellet debris. Protein concentration in the supernatant was determined by BCA assay. Equal protein amounts (20 µg per lane) were mixed with 5× SDS loading buffer, boiled at 100 °C for 10 min, and resolved on 10% SDS-PAGE gels (5% stacking). Proteins were transferred to PVDF membranes (wet transfer, 300 mA, 1 h). Membranes were blocked in 5% non-fat milk (in PBST) for 1 h at room temperature, then incubated overnight at 4 °C with primary antibodies (e.g. anti-TRIB3, anti–phospho-PERK, anti-p62, β-actin; see Key Resources). After washing (PBST 3 × 5 min), HRP-conjugated secondary antibodies were applied for 1 h at room temperature. Blots were washed again and developed using ECL substrate. Chemiluminescence images were captured on a digital imager. Band intensities were quantified with ImageJ and normalized to the β-actin loading control. This procedure follows standard immunoblotting protocols. Detailed information on antibodies used for western blotting is provided in Supplementary Table S3.

### Genotyping (Trib3 knockout)

Tail snips (~ 2 mm) from weanling mice were used for genomic DNA extraction (commercial kit; Biolinked, WE0186). PCR genotyping was performed using two primer pairs (F1/R1 and F2/R1 as listed in Supplementary Table S4) to distinguish the wild-type and mutant alleles. PCR products were separated on 1% agarose gels (150 V, 30 min). Wild-type alleles produce a 1602 bp band (PCR-I), whereas knockout alleles lack this band but yield a 546 bp product with the second primer set (PCR-II). All genotypes were confirmed by the expected band patterns.

### Quantification and statistical analysis

Quantitative data are presented as mean ± standard error of the mean (SEM). Sample sizes (n) and numbers of independent experiments are indicated in figure legends. Statistical analyses were performed using GraphPad Prism 9.0. For comparisons among multiple groups, one-way ANOVA followed by Tukey’s post hoc test was used to determine significance. For clinical score analysis, repeated-measures two-way ANOVA was used, with time as the within-subject (repeated) factor and treatment group as the between-subject factor. This approach has been widely applied for longitudinal EAE clinical score analysis [38]. A p value < 0.05 was considered statistically significant. Post hoc comparisons were performed as indicated in the figures (*P < 0.05, **P < 0.01, ***P < 0.001, ****P < 0.0001). All statistical tests were two-tailed. Exact p values and details of tests are reported in figure legends. All key experiments were reproduced at least three times with consistent results.

## Results

### Post-onset intermittent fasting (EAE_postIF) attenuates clinical disease and neuropathology

**Fig. 1:**
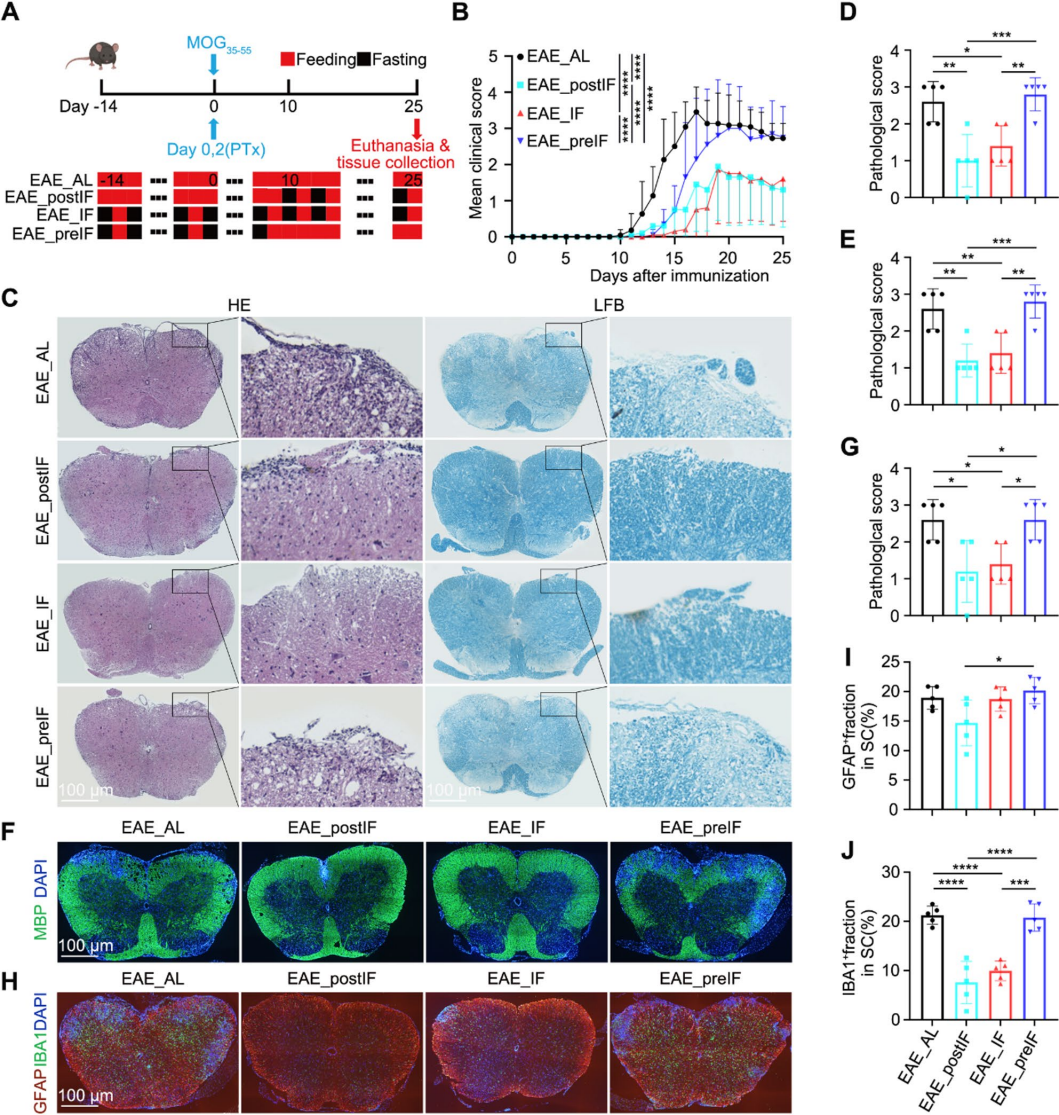
EAE_postIF and EAE_IF reduce clinical severity and CNS pathology in EAE. (**A**) Experimental design: female C57BL/6J mice were induced with MOG_35-55_ to establish the EAE model and randomly assigned to four groups: (1) EAE_AL: EAE induction, fed ad libitum throughout; (2) EAE_postIF: Immunized on day 0, ad libitum feeding until day 10, then IF every other day from day 10 until endpoint; (3) EAE_IF: Immunized on day 0, IF every other day from day -14 until endpoint; (4) EAE_preIF: Immunized on day 0, IF every other day from day -14 to day 10, followed by ad libitum feeding thereafter. Mice were euthanized at day 25. (**B**) Mean clinical scores (0-5 scale) over time (n = 10-12 per group) show that EAE_postIF and EAE_IF mice develop significantly milder disease than EAE_AL (two-way ANOVA: ****P < 0.0001). (**C**) Representative spinal cord sections (lumbar) at day 25: H&E staining (top) and LFB staining (bottom). EAE_AL and EAE_preIF cords show dense inflammatory infiltrates (H&E) and myelin loss (LFB); EAE_postIF and EAE_IF show reduced inflammation and preserved myelin. Scale bar = 100 μm. (**D–E**) Quantification of H&E pathology (**D**) and LFB demyelination (**E**) scores. EAE_postIF and EAE_IF have significantly lower lesion scores than EAE_AL (*P < 0.05, **P < 0.01; one-way ANOVA; n = 5). (**F**) MBP immunofluorescence of spinal cord (green, myelin); nuclei counterstained with DAPI (blue). As expected, MBP loss is severe in EAE_AL and EAE_preIF, whereas EAE_postIF and EAE_IF show stronger MBP signal (scale bar = 100 μm). (**G**) Quantification of MBP^+^ area or demyelination scores, confirming significant myelin preservation in EAE_postIF and EAE_IF (*P < 0.05,**P < 0.01; n = 5). (**H**) Representative IBA1 (green, microglia) and GFAP (red, astrocytes) IF in spinal cord (scale bar = 100 μm). (**I–J**) Percent area occupied by IBA1^+^ microglia and GFAP^+^ astrocytes, respectively. EAE_postIF and EAE_IF have significantly fewer activated glia than EAE_AL; GFAP is also lower in EAE_postIF vs EAE_preIF (**P< 0.01, ****P < 0.0001; n = 5). Data are mean ± SEM; statistics by one-way ANOVA with Tukey’s test. All experiments independently replicated 3 times

We compared groups of EAE mice that received no dietary intervention (EAE_AL), continuous IF (EAE_IF), IF starting on day 10 post-induction (EAE_postIF), or IF only prior to induction (EAE_preIF) (Fig. 1 A). Mice in the EAE_postIF and EAE_IF groups developed significantly milder disease, as reflected by lower daily clinical scores over time compared with EAE_AL or EAE_preIF mice (Fig. 1B), indicating that initiating IF at or shortly after onset slows EAE progression. This improvement persisted through the 25-day endpoint. By contrast, pre-induction fasting alone (EAE_preIF) showed only a transient early improvement that was not sustained at later stages. Consistently, the cumulative clinical scores were significantly lower in the EAE_postIF and EAE_IF groups, and their overall disease incidence also tended to be lower than that of EAE_AL mice (Fig.S1A and B). Notably, body weight trends and overall food/water intake were similar across groups (Fig. S2), ruling out generalized malnutrition. These findings align with prior reports that therapeutic fasting can reduce EAE severity.

Histological analysis of spinal cords at day 25 revealed marked differences consistent with the clinical scores. Hematoxylin/eosin (H&E) staining showed extensive inflammatory infiltrates in EAE_AL and EAE_preIF mice, whereas EAE_postIF and EAE_IF cords had markedly less parenchymal inflammation (Fig. 1 C and D). Likewise, Luxol Fast Blue (LFB) staining and MBP immunofluorescence demonstrated severe demyelination in EAE_AL and EAE_preIF mice, whereas EAE_postIF and EAE_IF groups preserved much more myelin (Fig. 1 C, F, S1C and D). Quantification of inflammation and demyelination (based on standard scoring) confirmed significantly lower pathology in EAE_postIF and EAE_IF versus EAE_AL (Fig. 1D and G). In parallel, markers of glial activation were reduced by EAE_postIF: immunofluorescent IBA1 (microglia) and GFAP (astrocytes) signals were significantly lower in the EAE_postIF and EAE_IF mice than in EAE_AL (Fig. 1H and J). Notably, GFAP was significantly lower in EAE_postIF than in EAE_preIF, suggesting that late IF was more effective at curbing astrocyte activation. Although the EAE_preIF regimen provided a transient improvement during early disease stages, its protective effects were not sustained once normal feeding resumed, indicating that intervention timing critically influences efficacy. Together, these data indicate that IF initiated at early clinical onset limits neuroinflammation and demyelination, consistent with reports that fasting regimens can promote remyelination and limit autoimmunity in EAE models.

### EAE_postIF does not significantly alter Th1/Th17/Treg proportions in CNS or periphery

**Fig. 2:**
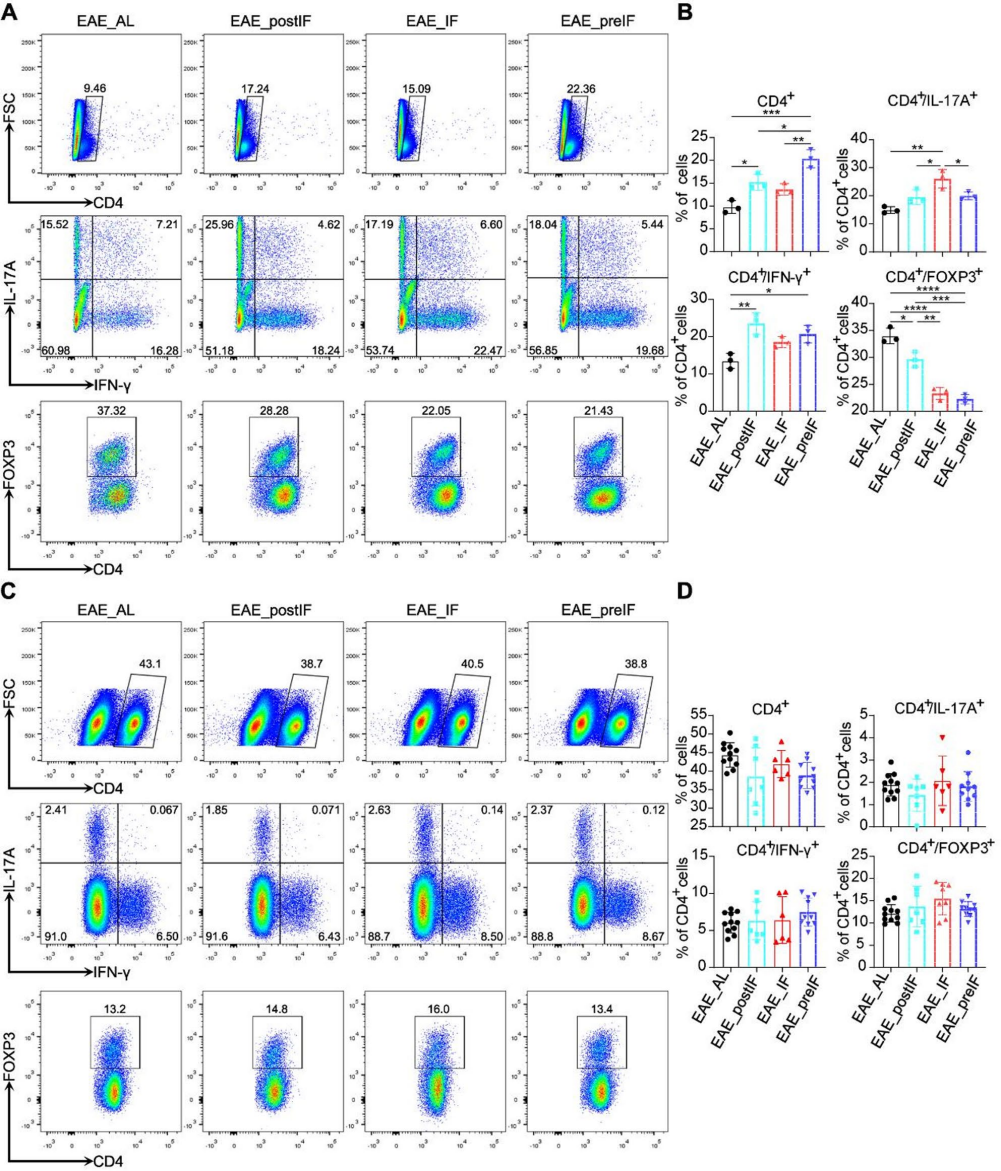
IF intervention causes no consistent changes in Th1/Th17/Treg composition in CNS or periphery.(**A**) Representative flow cytometry plots of spinal cord mononuclear cells. Cells were gated on CD4^+^ T lymphocytes and analyzed for IFN-γ, IL-17A, and FoxP3 expression. Percentages shown for IFN-γ^+^ (Th1), IL-17A^+^ (Th17) and FoxP3^+^ (Treg) fractions. (**B**) Quantification of CD4^+^IFN-γ^+^, CD4^+^IL-17A^+^, and CD4^+^FoxP3^+^ cells in the spinal cords of each group (EAE_AL, EAE_postIF, EAE_IF, and EAE_preIF). Data are presented as mean ± SEM (n = 5 per group, one-way ANOVA). (C–D) Analogous analysis on splenic CD4^+^ T cells. (**C**) Representative flow plots; (**D**) Quantification. Again, IF did not significantly change Th1, Th17, or Treg frequencies in spleen (one-way ANOVA). Data are mean ± SEM; analysis performed on n mice per group. Experiments independently replicated 3 times

To explore immune mechanisms, we used flow cytometry to profile CD4^+^ T cell subsets in spinal cord and spleen (Fig. 2). CD4⁺ T cells were gated from singlet lymphocytes and identified by intracellular IFN-γ, IL-17 A, and FOXP3 staining to define Th1, Th17, and Treg subsets (Fig S3). Among EAE groups, EAE_AL mice exhibited the highest clinical scores, indicating the most severe disease, yet surprisingly showed the lowest proportions of CD4⁺IFN-γ⁺ (Th1) and CD4⁺IL-17 A⁺ (Th17) cells and the highest CD4⁺FOXP3⁺ (Treg) fraction (Fig. 2A–B). In contrast, the EAE_preIF group, which had comparable clinical severity to EAE_AL at the endpoint, displayed higher overall CD4⁺ infiltration and elevated Th17 frequencies. Collectively, the relative proportions of Th1, Th17, and Treg cells did not show a consistent correlation with disease severity among the EAE groups (Fig. 2A and B). Similarly, spleen (peripheral) CD4^+^ T subsets were unchanged across diets (Fig. 2C and D). In particular, IF did not alter the overall CD4^+^ T cell frequency or skew differentiation toward Tregs or away from Th1/Th17 compared to EAE_AL. These results imply that the protective effect of EAE_postIF is not mediated by gross changes in peripheral or CNS CD4^+^ T cell subset frequencies, although previous human studies have shown that chronic intermittent fasting can shift circulating memory T cell populations in MS patients [39, 40]. In our model, local changes in other immune cell types or cytokine production may instead underlie the benefit of IF.

### EAE_postIF activates ER stress response and autophagy pathways in EAE spinal cord

**Fig. 3:**
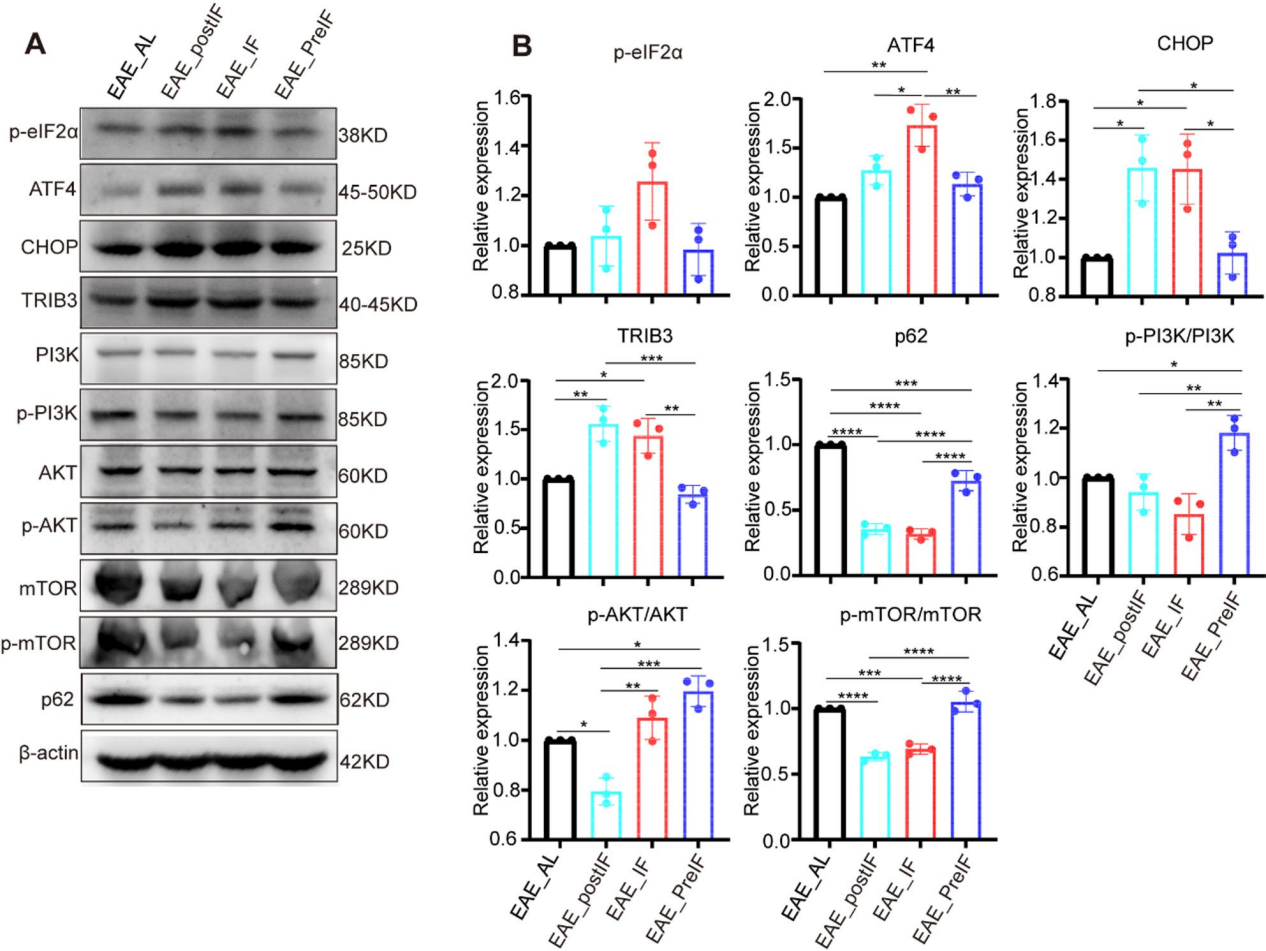
EAE_postIF and EAE_IF activate the PERK–ATF4–CHOP pathway and inhibits AKT/mTOR in EAE spinal cord. (**A**) Western blots of lumbar spinal cord lysates from EAE_AL, EAE_postIF, EAE_IF, and EAE_preIF groups (n = 3 per group). IF-treated EAE mice show increased levels of p-eIF2α, ATF4, CHOP, and TRIB3 (indicative of PERK pathway activation). Autophagy markers are consistent with mTOR inhibition: p62 is markedly reduced, and the ratios p-AKT/AKT and p-mTOR/mTOR are decreased. β-actin is loading control. (**B**) Densitometric quantification (normalized to β-actin or total protein) of the blots in (A). EAE_postIF and EAE_IF have significantly higher ATF4, CHOP, TRIB3, and lower p62, p-AKT, and p-mTOR than EAE_AL (*P < 0.05, **P < 0.01, ***P < 0.001; one-way ANOVA). These data indicate that EAE_postIF engages the ER stress response and promotes autophagy in EAE spinal cord. Experiments independently replicated 3 times

We next examined molecular pathways. Western blots of spinal cord lysates revealed that the PERK–eIF2α–ATF4–CHOP axis of the unfolded protein response (UPR) was strongly induced by IF. EAE_postIF and EAE_IF mice showed markedly higher levels of p-eIF2α, ATF4, CHOP, and the downstream stress target TRIB3 than EAE_AL or EAE_preIF mice (Fig. 3A and B). These changes indicate activation of the PERK branch of the UPR in the disease-affected spinal cord under fasting. Concurrently, markers of the PI3K–AKT–mTOR pathway were suppressed: both EAE_postIF and EAE_IF spinal cords had reduced p-AKT/AKT and p-mTOR/mTOR ratios, and a strong reduction of the autophagy substrate p62. The concomitant induction of PERK–ATF4–CHOP signaling and suppression of mTOR activity indicates enhanced autophagic flux in fasting mice. Indeed, mTOR inhibition is known to activate autophagy and has been reported to ameliorate EAE via neuroprotective autophagy induction [41–43]. Thus, post-onset IF appears to engage an ER stress–autophagy axis, consistent with the concept that metabolic stress can boost protein quality control and cell survival mechanisms in neuroinflammatory disease.

**Fig. 4:**
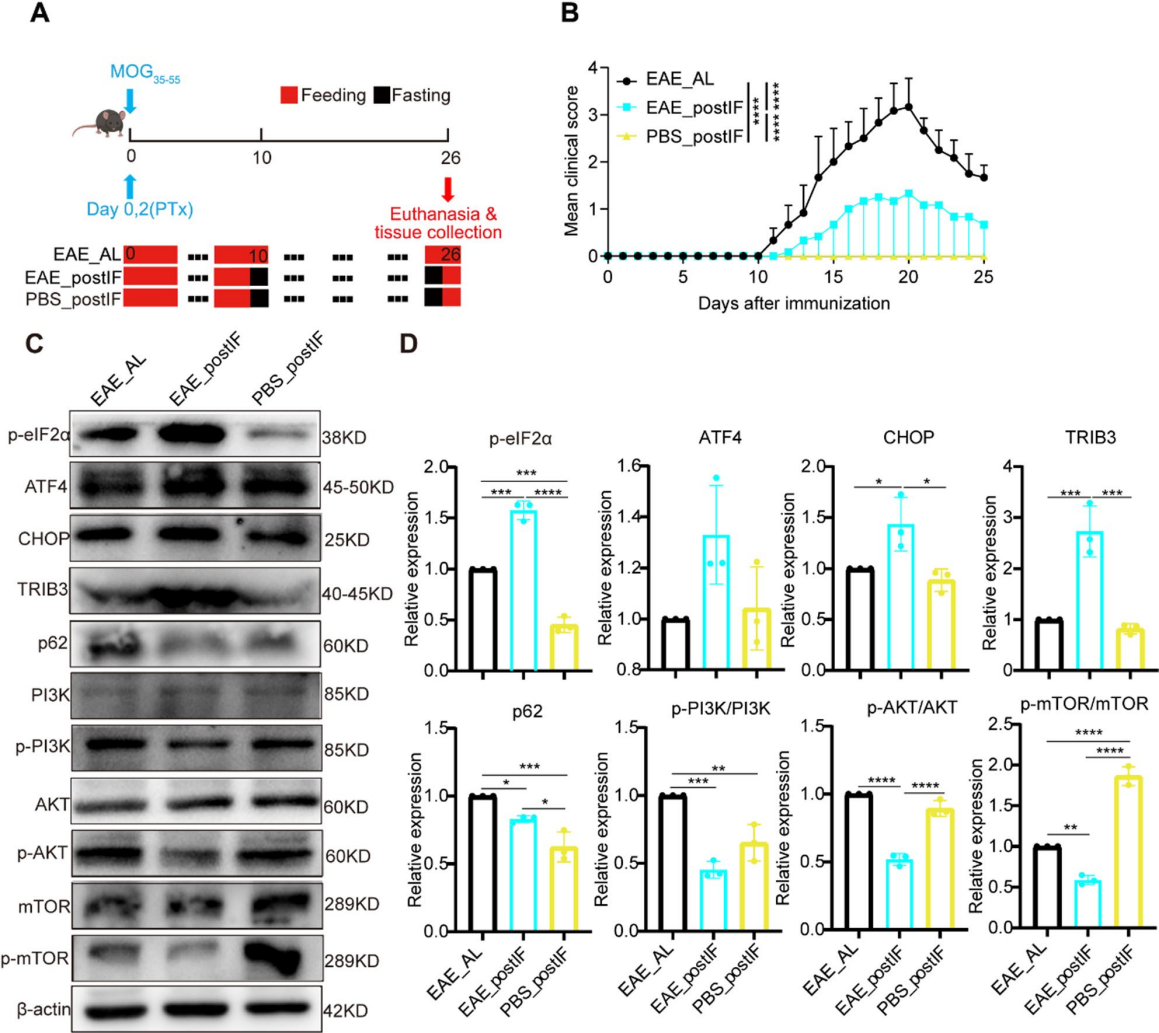
Effects of post-onset IF in EAE vs. healthy mice.(**A**) Experimental design: a control group (PBS_postIF) of non-immunized mice underwent the same fasting schedule as EAE_postIF. (**B**) Clinical scores (n = 6). EAE_postIF mice exhibited significantly lower clinical scores compared with EAE_AL (****P< 0.0001). (**C**) Western blots of PERK/mTOR pathway proteins from spinal cords of EAE_AL (EAE without IF), EAE_postIF, and PBS_postIF (n = 3 each). (**D**) Quantification of (C). Compared to EAE_AL, PBS_postIF mice have significantly lower p-eIF2α (**P < 0.01) and no increase in CHOP/TRIB3, indicating that IF alone (no EAE) does not activate PERK signaling. Both EAE_postIF and PBS_postIF have lower p-PI3K than EAE_AL (*P < 0.05). Only EAE_postIF shows significant reductions in p-AKT and p-mTOR (***P < 0.001 vs. EAE_AL), whereas PBS_postIF does not. Conversely, p-mTOR is higher in PBS_postIF than EAE_AL (***P < 0.001 vs. EAE_AL), suggesting mTOR pathway is maintained under IF without disease. Thus, fasting in healthy mice does not induce the same stress response seen in EAE

To test whether fasting alone (without disease) similarly triggers these pathways, we compared EAE_postIF mice to non-immunized mice under the same IF regimen (PBS_postIF) (Fig. 4 A). Body weight, water, and food intake were monitored throughout the experiment, and no significant differences were observed between PBS_postIF and EAE_postIF mice (Fig. S4), indicating that fasting was well-tolerated in both groups. PBS_postIF mice showed no clinical signs (flat score, Fig. 4B) and minimal pathology (Fig. S5), indicating that fasting alone did not induce significant pathological changes in healthy animals.

In spinal cords, PBS_postIF mice exhibited lower PERK pathway activation than EAE_postIF mice: p-eIF2α, CHOP, and TRIB3 were all significantly reduced in PBS_postIF compared with EAE_postIF. This indicates that ER stress signaling was not activated under fasting conditions in non-EAE animals relative to diseased mice. Correspondingly, p62 expression was lowest in EAE_postIF, suggesting that autophagy was specifically engaged in the disease context. Together, these findings suggest that activation of the PERK–autophagy axis by intermittent fasting depends on the presence of autoimmune stress.

### TRIB3 is required for EAE_postIF mediated protection

**Fig. 5:**
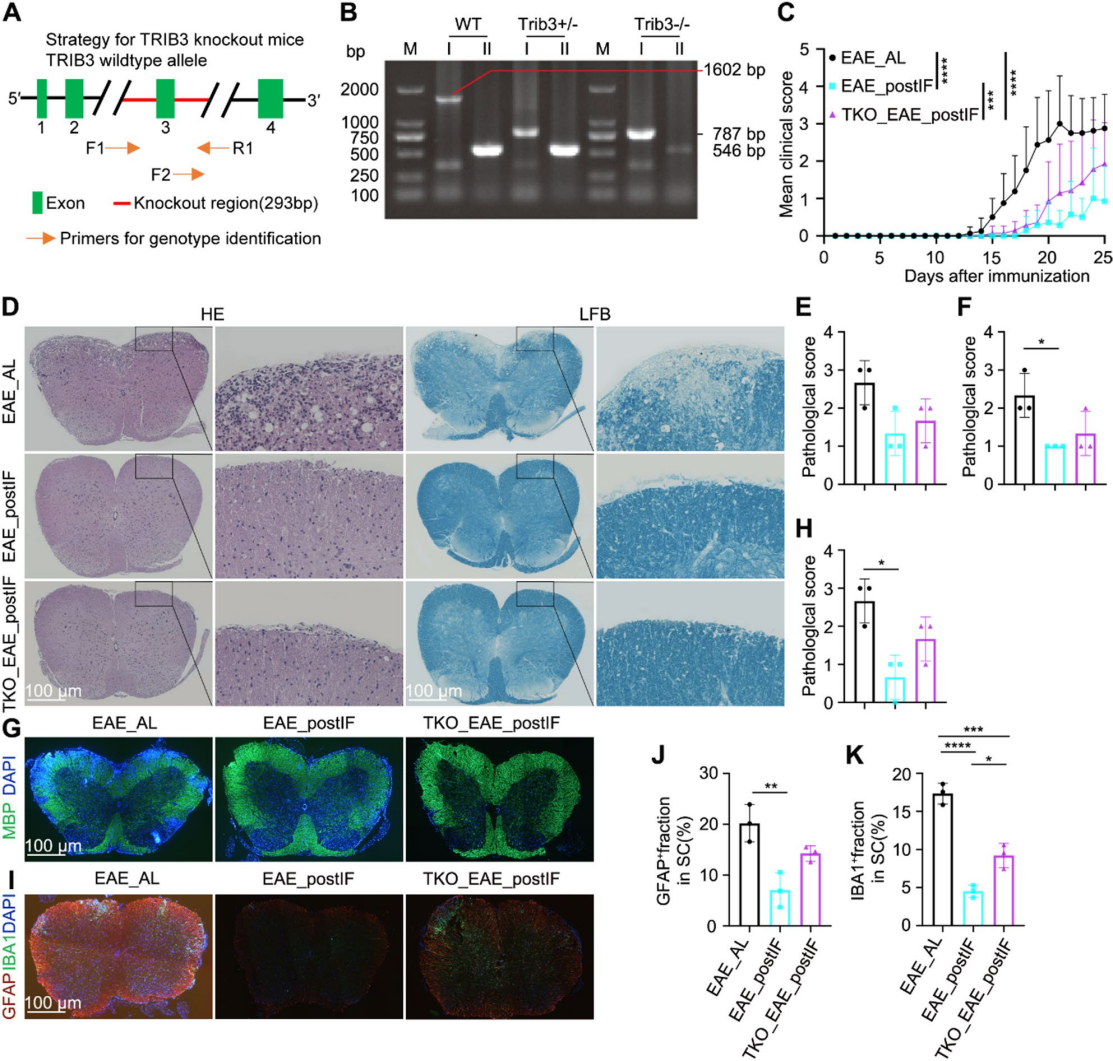
TRIB3 deletion diminishes the neuroprotective effect of IF in EAE. (**A**) Schematic of Trib3^–/–^ knockout strategy (exon 3 deletion by CRISPR). (**B**) Genotyping PCR of mouse tail DNA (PCR-I amplifies intact Trib3, PCR-II only amplifies deletion allele) confirms TRIB3KO genotype. (**C**) Clinical scores (n = 8 per group): IF significantly reduces symptoms in wild-type mice (EAE_postIF vs. EAE_AL, ***P < 0.001). TRIB3KO mice under IF (TKO_EAE_postIF) have worse scores than IF-treated wild type (***P < 0.001), though still better than EAE_AL (*P < 0.05). (**D–F**) Representative H&E (D, left) and LFB (D, right) spinal cord sections for EAE_AL, EAE_postIF, and TKO_EAE_postIF. (**E**) Inflammation scores from H&E and (**F)** demyelination scores from LFB. EAE_postIF shows reduced pathology vs. EAE_AL, whereas TRIB3KO_IF mice have scores similar to EAE_AL (one-way ANOVA, *P < 0.05 compared to EAE_AL; n = 3). (**G**) MBP IF in spinal cord (green); (**H**) MBP demyelination score. EAE_postIF maintains MBP whereas TRIB3KO_IF lost myelin protection (*P < 0.05 vs. EAE_AL, n = 3). (**I**) IBA1 (green) and GFAP (red) IF images (scale bar = 100 μm). (**J–K**) Quantified area fraction of GFAP^+^ and IBA1^+^ staining. EAE_postIF has significantly lower IBA1 and GFAP than EAE_AL. TKO_EAE_postIF shows higher IBA1 than EAE_postIF (***P < 0.001) but lower than EAE_AL; GFAP in TRIB3KO_IF is intermediate (n = 3). Overall, TRIB3 deletion partially abrogates the reduction in neuroinflammation and demyelination normally conferred by IF. Experiments independently replicated 3 times

Given TRIB3’s role linking PERK to AKT/mTOR signaling, we tested the effect of Trib3 deletion. Figure 5 A and B shows the genotyping results of TRIB3 knockout mice. Figure 5 A illustrates the knockout strategy, where exon 3 of the TRIB3 gene was deleted using CRISPR. Genotyping PCR confirmed the successful deletion of the TRIB3 gene, with wild-type (WT) and knockout (KO) alleles clearly differentiated by the PCR primers used. Figure 5B presents the PCR amplification results, confirming the presence of the TRIB3 knockout genotype in the homozygous knockout mice. Consistently, Western blot analysis further verified the absence of TRIB3 protein expression in these mice (Fig S6A). Trib3^−/−^ mice underwent EAE induction and the same post-onset IF schedule (TKO_EAE_postIF). These mice still benefited from IF relative to untreated controls: their clinical scores were significantly lower than EAE_AL but significantly higher than IF-treated wild-type EAE_postIF mice (Fig. 5 C). Consistently, the cumulative clinical scores showed the same trend, and disease incidence also tended to be lower in TKO_EAE_postIF mice compared with EAE_AL (Fig. S6B and C). Body weight, water, and food intake were monitored and showed no significant differences between TKO_EAE_postIF and EAE_postIF groups, indicating similar tolerability to the fasting regimen (Fig. S7). In other words, IF still reduced symptoms without TRIB3, but less effectively. Histopathology reflected this partial loss of protection. The extensive myelin preservation seen in EAE_postIF was lost in TKO_EAE_postIF: H&E and LFB staining showed demyelination in TRIB3KO mice comparable to EAE_AL, and quantitative myelin scores were significantly worse in TRIB3KO than in EAE_postIF (no different from EAE_AL) (Fig. 5D and F). MBP immunofluorescence staining showed that EAE_postIF mice preserved more myelin integrity compared to TKO_EAE_postIF mice, which exhibited significant myelin loss (Fig. 5G and H). Similarly, IBA1^+^ microglia were elevated in TKO_EAE_postIF versus EAE_postIF (though still below EAE_AL). GFAP^+^ astrocytes followed the same trend (Fig. 5I, J, S6D and E). These data indicate that TRIB3 mediates a substantial part of IF’s neuroprotective effect.

**Fig. 6:**
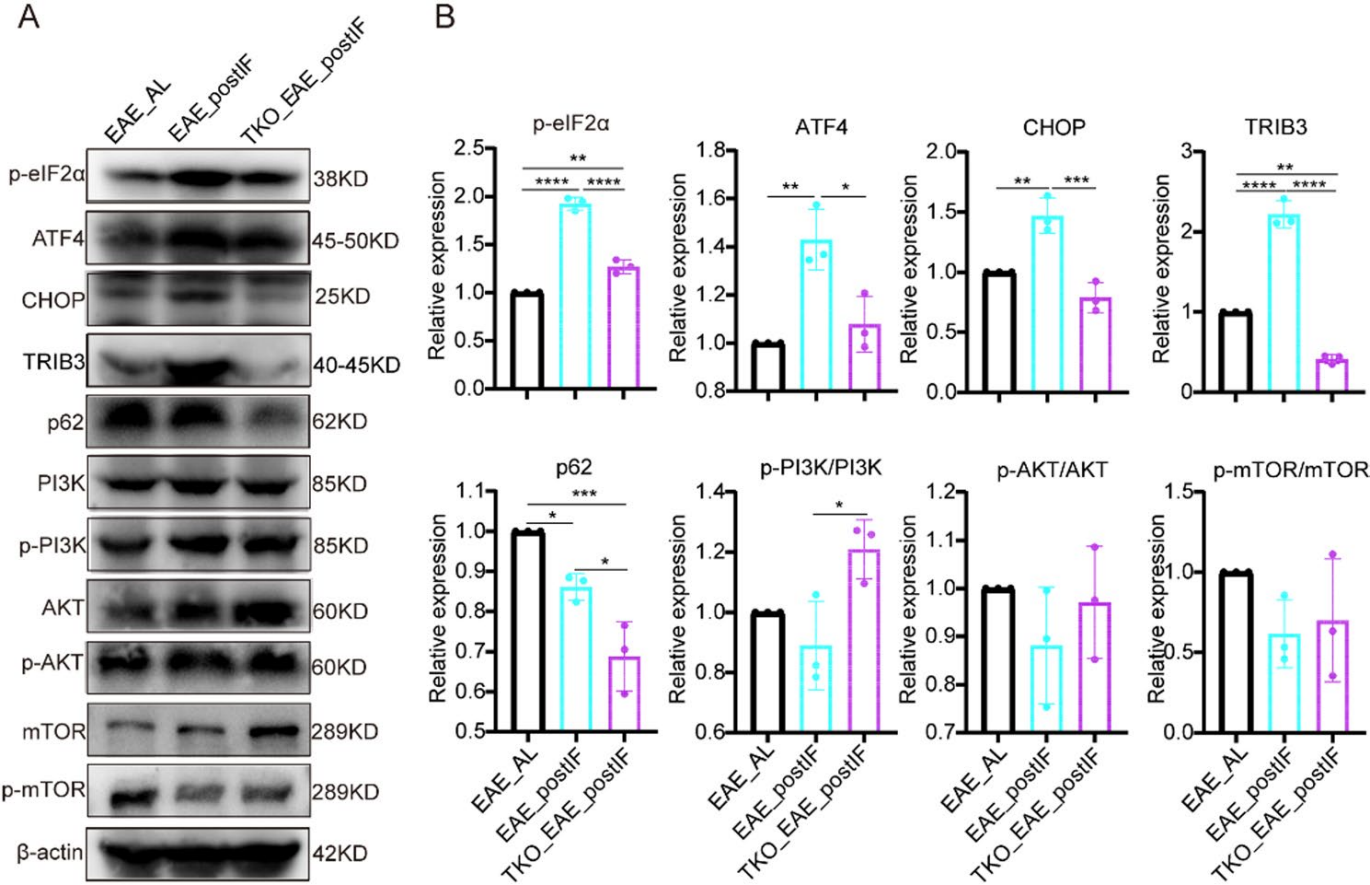
Loss of TRIB3 impairs PERK signaling and reactivates AKT/mTOR under IF.(**A**) Western blots of spinal cord extracts from EAE_AL, EAE_postIF, and TKO_EAE_postIF (n = 3 each). Compared to EAE_postIF, TRIB3KO mice show reduced p-eIF2α, ATF4, and CHOP (weakened PERK response) and increased p-AKT and p-mTOR (loss of IF-induced inhibition). p62 remains low. (**B**) Quantification of (A). Relative to EAE_postIF, TKO_EAE_postIF has significantly lower p-eIF2α, ATF4, CHOP (*P < 0.05) and higher p-AKT, p-mTOR (*P < 0.05). These shifts imply that without TRIB3, the adaptive ER stress/autophagy program of IF is blunted, consistent with the intermediate clinical phenotype

Western blots confirmed that loss of TRIB3 blunted the IF-induced UPR/autophagy signals. TKO_EAE_postIF cords showed lower p-eIF2α, ATF4, and CHOP than EAE_postIF (consistent with a weaker PERK response). Conversely, these mice had higher p-AKT and p-mTOR levels than IF-treated wild-type (reflecting disinhibition of AKT/mTOR by loss of TRIB3). Paradoxically, p62 remained low in the TRIB3KO group (Fig. 6 A and B), suggesting autophagy was still active despite mTOR signaling. This may reflect compensatory regulation: multiple pathways (beyond TRIB3) can modulate autophagy. Overall, TRIB3 deletion limited the ability of IF to engage the PERK-dependent stress response and to suppress AKT/mTOR. These molecular changes correlate with the intermediate clinical phenotype, supporting our model that TRIB3 is a key node through which IF influences EAE progression.

### Post-onset time-restricted feeding (EAE_postTR) and mild caloric restriction (EAE_postCR) fail to attenuate EAE severity

**Fig. 7:**
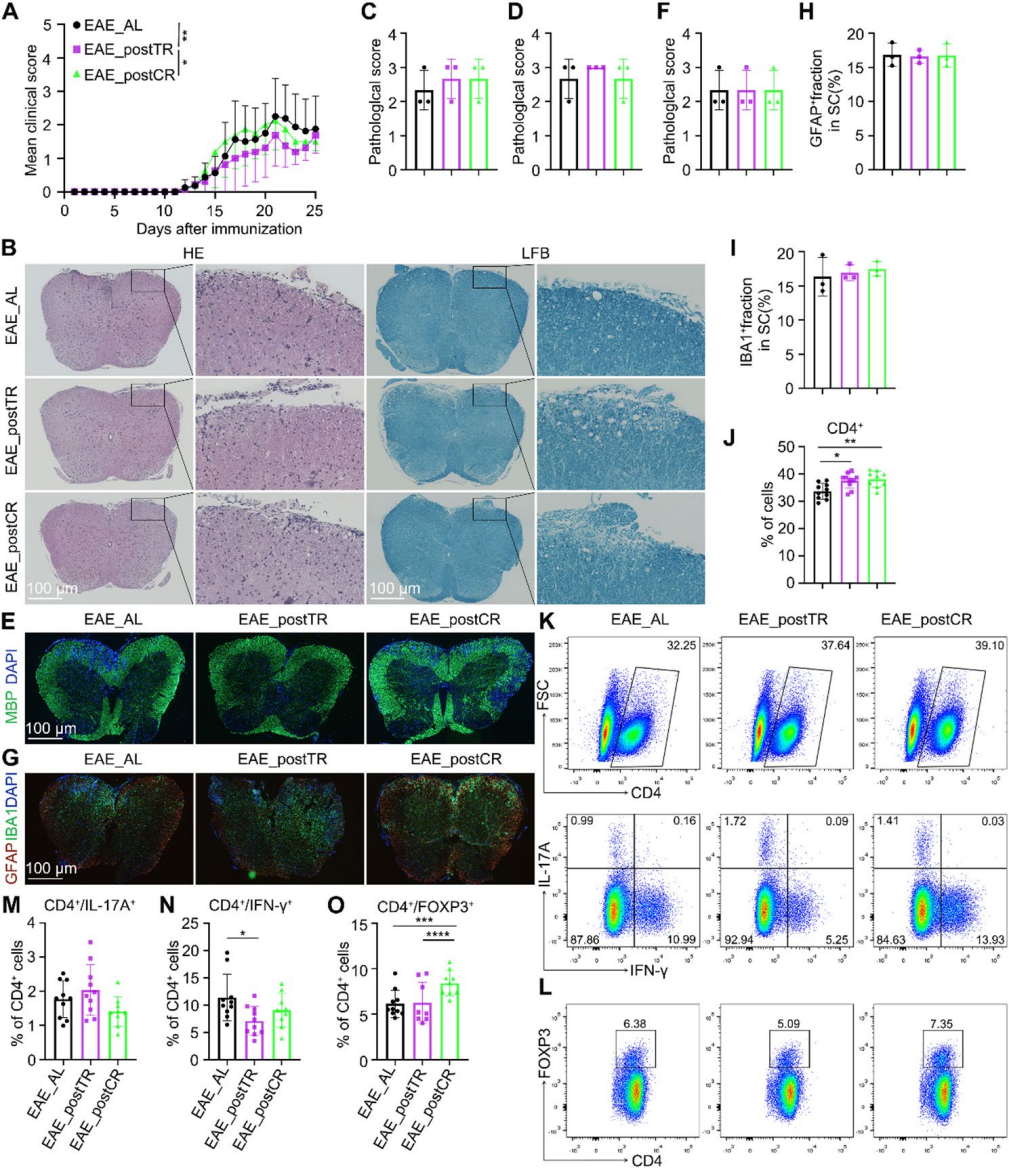
Time-restricted feeding (TR) or moderate caloric restriction (CR) do not improve EAE. (**A**) Clinical scores for mice receiving EAE_AL, 16h:8h time-restricted feeding (TR), or 70% caloric restriction (CR) (n = 6). Neither TR nor CR significantly lowers scores compared to EAE_AL (two-way ANOVA). (**B**) Representative spinal cord H&E images; (**C**) H&E inflammation scores; (**D**) LFB demyelination scores. There is no statistical difference among groups (one-way ANOVA; n = 5). (**E**) MBP IF images (green) and (**F**) MBP demyelination scores. All groups show similar MBP loss (n = 5). (**G**) IBA1/GFAP IF (scale 100 μm) and (**H–I**) quantification of IBA1^+^ and GFAP^+^ area fractions. Microglial and astrocyte activation are comparable in EAE_AL, TR, and CR (n = 5). (**J**) Percentage of CD4^+^ T cells in splenocytes by flow; (**K**) Representative flow plots of IFN-γ/IL-17A staining on CD4^+^ cells; (**L**)Representative flow plots of FOXP3 staining on CD4^+^ cells ; (**M**) The percentage of IFN-γ⁺ cells among CD4⁺ T cells was measured by flow cytometry; (**N**) The percentage of IL-17A^+^ cells among CD4⁺ T cells was measured by flow cytometry; (**O**) The percentage of FOXP3^+^ cells among CD4⁺ T cells was measured by flow cytometry

Finally, we asked whether less extreme feeding schedules could substitute for IF. Mice were subjected to either a 16 h : 8 h daily time-restricted feeding (TR) regimen or to a moderate CR diet (70% of ad libitum) beginning at day 10. Neither intervention significantly ameliorated EAE. Clinical scores in the EAE_postTR and EAE_postCR groups were statistically indistinguishable from EAE_AL (Fig. 7A), indicating minimal therapeutic effect. Consistently, cumulative clinical scores and disease incidence also showed no significant differences among the three groups (Fig. S8A and B). Body weight, water, and food intake were also monitored and showed no significant differences between the EAE_postTR, EAE_postCR, and EAE_AL groups (Fig. S9).

Likewise, histological analyses showed no reduction in spinal cord inflammation or demyelination: H&E, LFB, and MBP staining patterns were comparable across EAE_AL, EAE_postTR, and EAE_postCR (Fig. 7B–F). Microglial (IBA1) and astrocyte (GFAP) activation markers also showed no significant differences (Fig. 7G–I, S8C and D). In the spleen, EAE_postTR modestly increased total CD4^+^ counts (as expected under feeding restriction) and slightly reduced the percentage of CD4^+^IFN-γ^+^ Th1 cells, but had no consistent effect on Th17 or Treg fractions (Fig. 7J–O). EAE_postCR produced even smaller immunological changes.

Thus, unlike prolonged alternate-day fasting regimens, these milder interventions failed to confer protection in the EAE model. This indicates that the magnitude and temporal parameters of energy restriction constitute critical determinants for engaging neuroprotective mechanisms.

## Discussion

In this study, we systematically compared different dietary interventions in the MOG-induced EAE model and found that intermittent fasting (IF) initiated after disease onset (i.e., during the early stage of disease progression) significantly delayed disease progression. Compared with the EAE_AL group, EAE_postIF reduced clinical scores, alleviated neuroinflammation, and preserved myelin integrity. While the effect was slightly less pronounced than EAE_IF, EAE_postIF is of greater translational relevance, as it reflects a realistic clinical scenario where patients begin treatment only after symptom onset. Notably, the EAE_preIF regimen (fasting from two weeks before immunization until disease onset, followed by ad libitum feeding) produced a transient early improvement but failed to maintain long-term protection once normal feeding resumed. Previous studies have demonstrated that long-term pre-induction fasting improves EAE outcomes [9, 16, 44]. However, our results highlight the critical importance of intervention timing: short-term IF starting after disease onset still conferred significant benefits, whereas pre-induction fasting failed to show any therapeutic effect. This suggests that metabolic interventions are most effective when aligned with the early immunological phase of disease progression, where they can modulate the proinflammatory cascade and prevent downstream neurodegeneration.

In contrast, neither EAE_postTR nor EAE_postCR yielded comparable therapeutic effects. This discrepancy may be attributed to insufficient metabolic stress imposed by these milder interventions, which failed to activate key protective pathways such as the PERK signaling axis or autophagy. We demonstrated that EAE_postIF robustly activated PERK and its downstream markers (p-eIF2α, ATF4, CHOP), in agreement with recent studies showing that PERK plays a neuroprotective role in EAE and MS by enhancing cellular stress tolerance and limiting ER burden [17, 20, 45]. Additionally, PERK signaling promotes alternative macrophage polarization and mitochondrial metabolism [46], supporting the view that PERK acts at the intersection of metabolic and immune homeostasis in neuroinflammation. Our findings further suggest that the therapeutic benefit of intermittent fasting initiated after disease onset cannot be explained solely by reduced caloric intake. Both mild caloric restriction and time-restricted feeding failed to reproduce the effects of intermittent fasting, indicating that fasting-specific metabolic or stress-adaptation pathways may underlie its unique efficacy.

Alongside PERK activation, we observed significant upregulation of autophagy-related proteins, suggesting that IF engages both proteostasis and cytoprotection through the PERK–autophagy axis. PERK can induce autophagy via ATF4/CHOP-dependent transcriptional upregulation or indirect inhibition of mTORC1. Our findings align with prior reports that link mTOR activation to exacerbated inflammation in MS, while mTOR inhibition (e.g., via rapamycin) protects against disease progression [47]. The increase in autophagic flux likely facilitates the clearance of damaged organelles and misfolded proteins, reducing neurotoxic stress and supporting neural repair mechanisms.

A key mechanistic insight from our study involves TRIB3, a stress-induced pseudokinase regulated by ATF4/CHOP and downstream of PERK. TRIB3 deletion partially abrogated the neuroprotective effect of IF and led to exacerbated disease severity, indicating that TRIB3 is essential for mediating the therapeutic effects of IF. Mechanistically, TRIB3 inhibits AKT phosphorylation, thereby suppressing the mTOR pathway and promoting autophagy. Loss of TRIB3 resulted in sustained AKT/mTOR activation and impaired autophagy, consistent with previous reports demonstrating that TRIB3 disruption leads to loss of proteostasis and heightened cellular stress [26, 48]. These findings suggest that TRIB3 serves as a molecular bridge between PERK signaling and mTOR-dependent metabolic regulation, orchestrating cellular adaptation to inflammatory and metabolic stress.

From a translational perspective, our findings support the therapeutic potential of IF in MS and other autoimmune diseases. Clinical studies have already reported that intermittent energy restriction can reshape the gut microbiome and reduce inflammatory markers in MS patients [16, 49]. IF offers a non-pharmacologic, accessible, and cost-effective intervention that can be readily implemented in patient care. Nevertheless, its clinical application requires careful optimization, as factors such as nutritional status, comorbidities, and long-term adherence may influence efficacy. The present study used an acute EAE model and TRIB3 knockout mice, which limit the generalizability of findings. Future work should employ cell-specific TRIB3 deletions and evaluate IF in chronic or relapsing-remitting models. Furthermore, clinical trials are needed to determine the optimal IF regimen (e.g., fasting duration, frequency, and composition of feeding periods) to maximize therapeutic efficacy while minimizing adverse effects.

It should be noted that this study used only female C57BL/6 mice to ensure a consistent EAE phenotype. Because sex may influence immune and metabolic responses [29, 30], future studies will include male mice to determine whether post-onset intermittent fasting produces sex-specific effects on disease outcomes. Another limitation of this study is the absence of a non-EAE ad libitum control (PBS_AL), which prevents us from determining whether intermittent fasting alone affects ER stress or autophagy signaling under normal physiological conditions. Our current findings therefore specifically describe the modulation of the PERK–autophagy pathway by IF within the context of autoimmune neuroinflammation. It should also be noted that our study did not determine the specific CNS cell types in which TRIB3 exerts its effects. While TRIB3 upregulation was evident at the tissue level, its precise cellular localization remains to be clarified. Future work using cell-type–specific approaches will be valuable to define which neuronal or glial populations mediate the TRIB3-dependent effects of intermittent fasting. Collectively, these limitations highlight areas for further investigation to better define the broader physiological and mechanistic implications of intermittent fasting in neuroinflammatory disease.

In summary, we demonstrate that intermittent fasting, when initiated during the early symptomatic phase of EAE, alleviates disease progression by activating PERK signaling and autophagy in a TRIB3-dependent manner. Our findings position TRIB3 as a key regulator linking metabolic and stress response pathways and underscore the importance of precise timing in dietary interventions. These insights provide a rationale for future development of metabolism-targeted therapies for MS and potentially other neuroinflammatory disorders.

## Supplementary Information

Supplementary Material 1. Supplementary Material 1 includes Figures S1–S9 and Tables S1–S4.

Supplementary Material 2. Supplementary Material 2 includes original data supporting the main figures, including full uncropped gels and blots, as well as other raw experimental data.

## Data Availability

No datasets were generated or analysed during the current study.

## Abbreviations

MS: Multiple sclerosis
EAE: Experimental autoimmune encephalomyelitis
MOG: Myelin oligodendrocyte glycoprotein
CNS: Central nervous system
IF: Intermittent fasting
EAE postIE: Post-onset intermittent fasting group
EAE_IF: Continuous intermittent fasting group
EAE_preIF: Pre-induction intermittent fasting group
PBS_postIF: Post-onset intermittent fasting in non-immunized mice
EAE_postTR: Post-onset time-restricted feeding group
EAE_postCR: Post-onset caloric restriction group
PERK: PKR-like endoplasmic reticulum kinase
UPR: Unfolded protein response
ER: Endoplasmic reticulum
ATF4: Activating transcription factor 4
CHOP: C/EBP homologous protein (DDIT3)
TRIB3: Tribbles homolog 3
mTOR: Mechanistic target of rapamycin
PI3K: Phosphatidylinositol 3-kinase
AKT: Protein kinase B
p62/SQSTM1: Sequestosome 1
MBP: Myelin basic protein
GFAP: Glial fibrillary acidic protein
IBA1: Ionized calcium-binding adapter molecule 1
H&E: Hematoxylin and eosin
LFB: Luxol Fast Blue
DAPI: 4′,6-diamidino-2-phenylindole
CFA: Complete Freund’s adjuvant
PTX: Pertussis toxin
PCR: Polymerase chain reaction
IP: Intraperitoneal injection
SEM: Standard error of the mean
ANOVA: Analysis of variance
FACS: Fluorescence-activated cell sorting
IFN-γ: Interferon gamma
IL-17A: Interleukin 17A
FoxP3: Forkhead box P3
Treg: Regulatory T cell
Th1: T helper 1 cell
Th17: T helper 17 cell
