# Supplementary material for: Post-onset intermittent fasting attenuates neuroinflammation and demyelination via a TRIB3–PERK–autophagy axis in an EAE model of multiple sclerosis
Source: J Neuroinflammation. 2025 Nov 27;22:301. doi: 10.1186/s12974-025-03640-y (PMC12752437; doi:10.1186/s12974-025-03640-y)
Supplement: Supplementary file 1 — Supplementary Material 1. Supplementary Material 1 includes Figures S1–S9 and Tables S1–S4. [file 12974_2025_3640_MOESM1_ESM.docx]

**Supplementary Information for**

**Post****-onset intermittent fasting attenuates neuroinflammation and demyelination via a** **TRIB3****–PERK–autophagy axis in an EAE model of multiple sclerosis**

*Weitai He¹^†^, Xueli Liu¹^†^, Di Wang²^†^, Ye Gong¹, Tingting Cui¹, Xin Zhang¹, Pei Li¹, Xiaoli Ding¹, Luting Yang¹, Qian Zhang¹, Yang Yang¹, Xiaochang Xue¹, Lin Shi³*, Yaling Zhang¹*, Yaping Yan¹**

This file includes:

Figs. S1 to S9

Tables S1 to S4

**
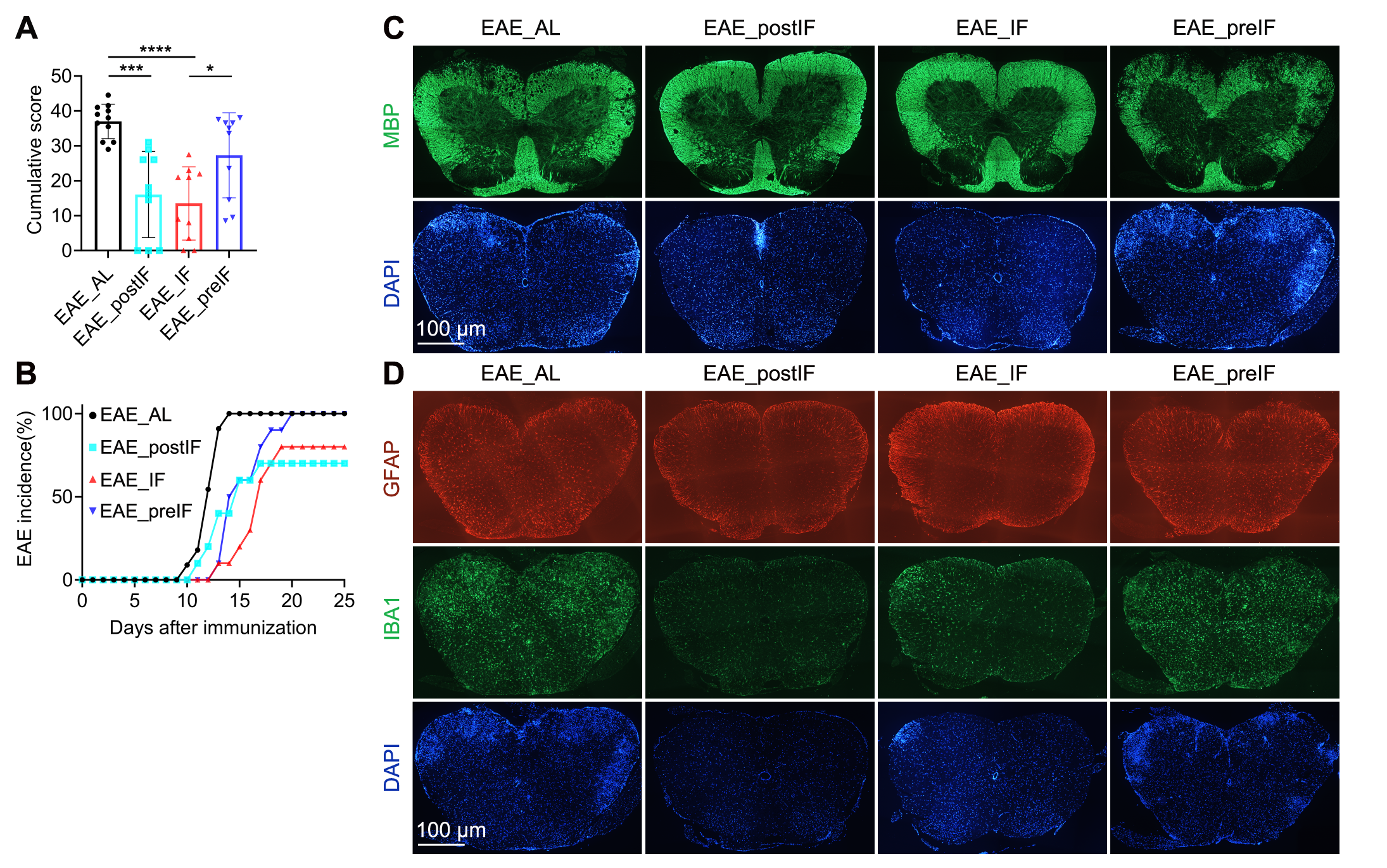
Figure S1. Spinal cord demyelination and glial activation after post-onset IF intervention. Related to Figure 1.** **(A)** Cumulative clinical scores of mice in EAE_AL, EAE_postIF, EAE_IFand EAE_preIF groups. Data represent mean ± SEM. Statistical analysis was performed by one-way ANOVA followed by Tukey’s post hoc test. **(B)** EAE incidence over time during disease progression in the same groups. **(C)** Representative immunofluorescence images of MBP (green) and DAPI (blue) staining in lumbar spinal cord sections from EAE_AL, EAE_postIF, EAE_IF, and EAE_preIF groups. **(D)** Representative immunofluorescence images of GFAP (red), IBA1 (green), and DAPI (blue) staining in the same groups. IF interventions reduced microglial (IBA1⁺) and astrocytic (GFAP⁺) activation while preserving MBP⁺ myelin integrity. scale bar = 100 μm.

**
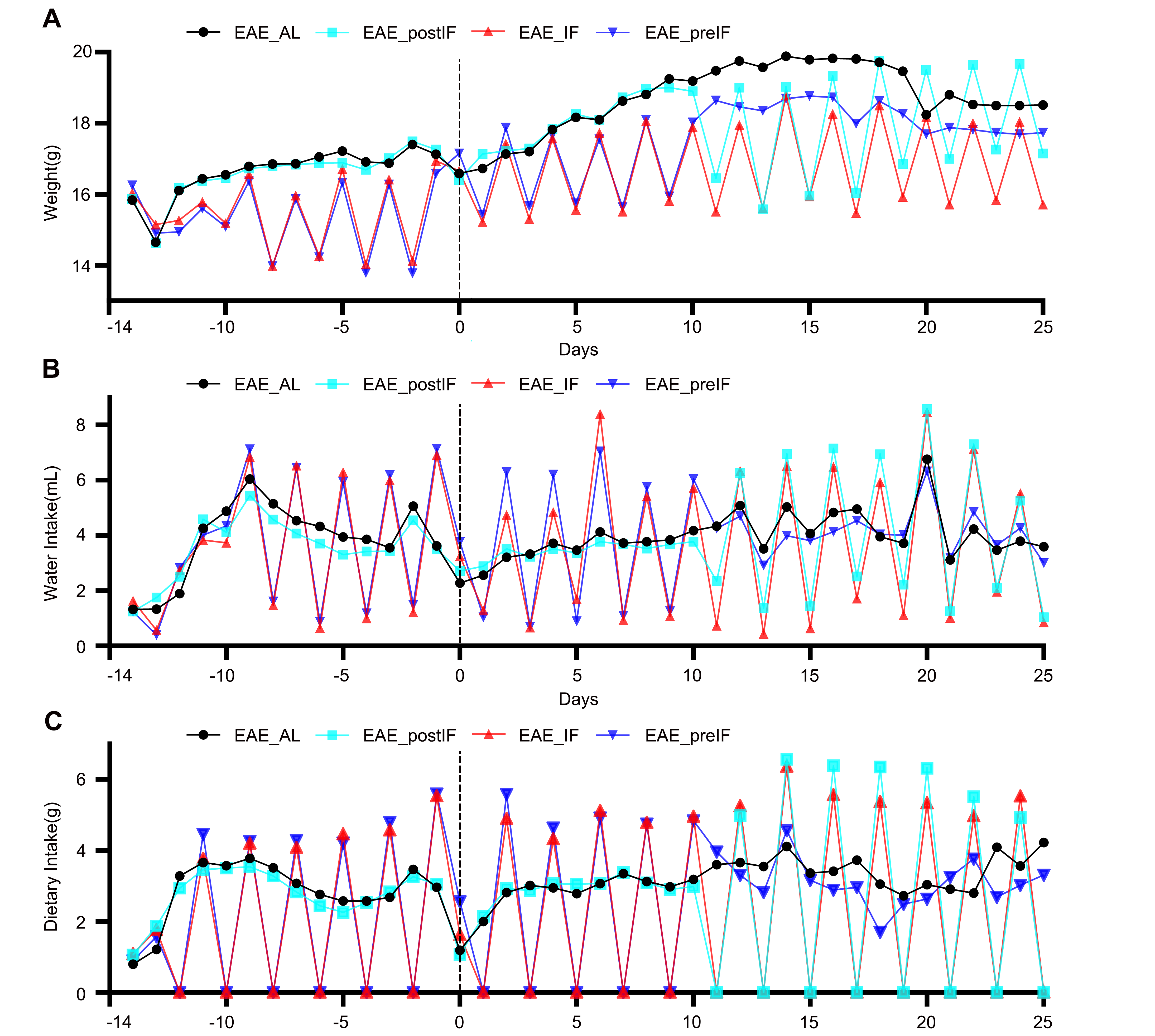
Figure S2. Body weight, water, and food intake in EAE mice under post-onset IF. Related to Figure 1.**(A–C) Mean body weight (A), water consumption (B), and food consumption (C) over time in EAE_AL, EAE_postIF, EAE_IF, and EAE_preIF groups (n = 10 - 12 per group). All groups show slight weight loss post-immunization but no significant differences; intake was similar across diets. Data are mean ± SEM.


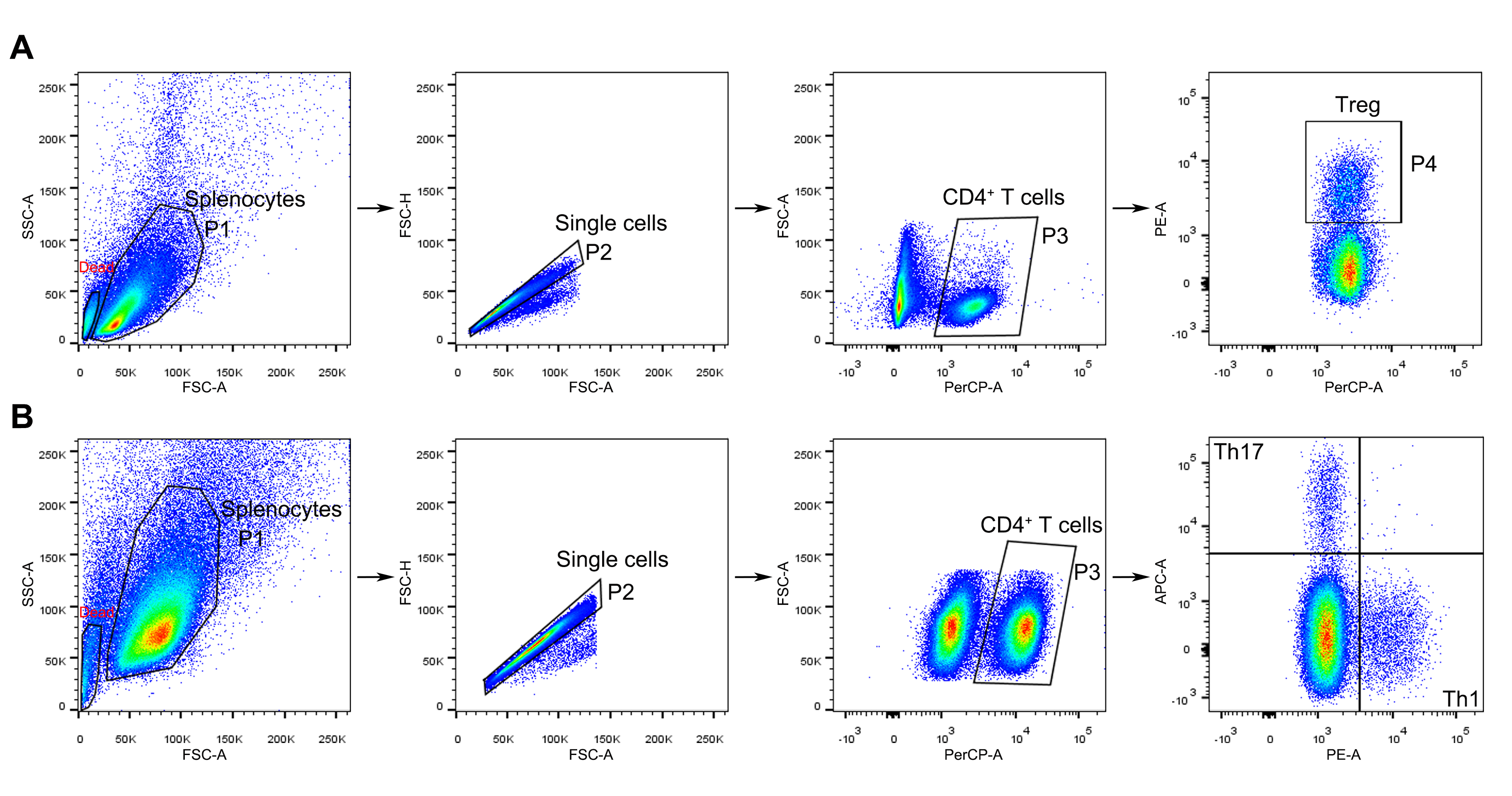
**Figure S3. Gating strategy for flow cytometric analysis of CD4⁺ T cell subsets. Related to Figure 2.** **(A)** Sequential gating for Treg cells (FSC-A/SSC-A → FSC-A/FSC-H → CD4⁺ → FOXP3⁺). **(B)** Gating for Th1 (CD4⁺IFN-γ⁺) and Th17 (CD4⁺IL-17A⁺) cells (PE-A vs APC-A).

**
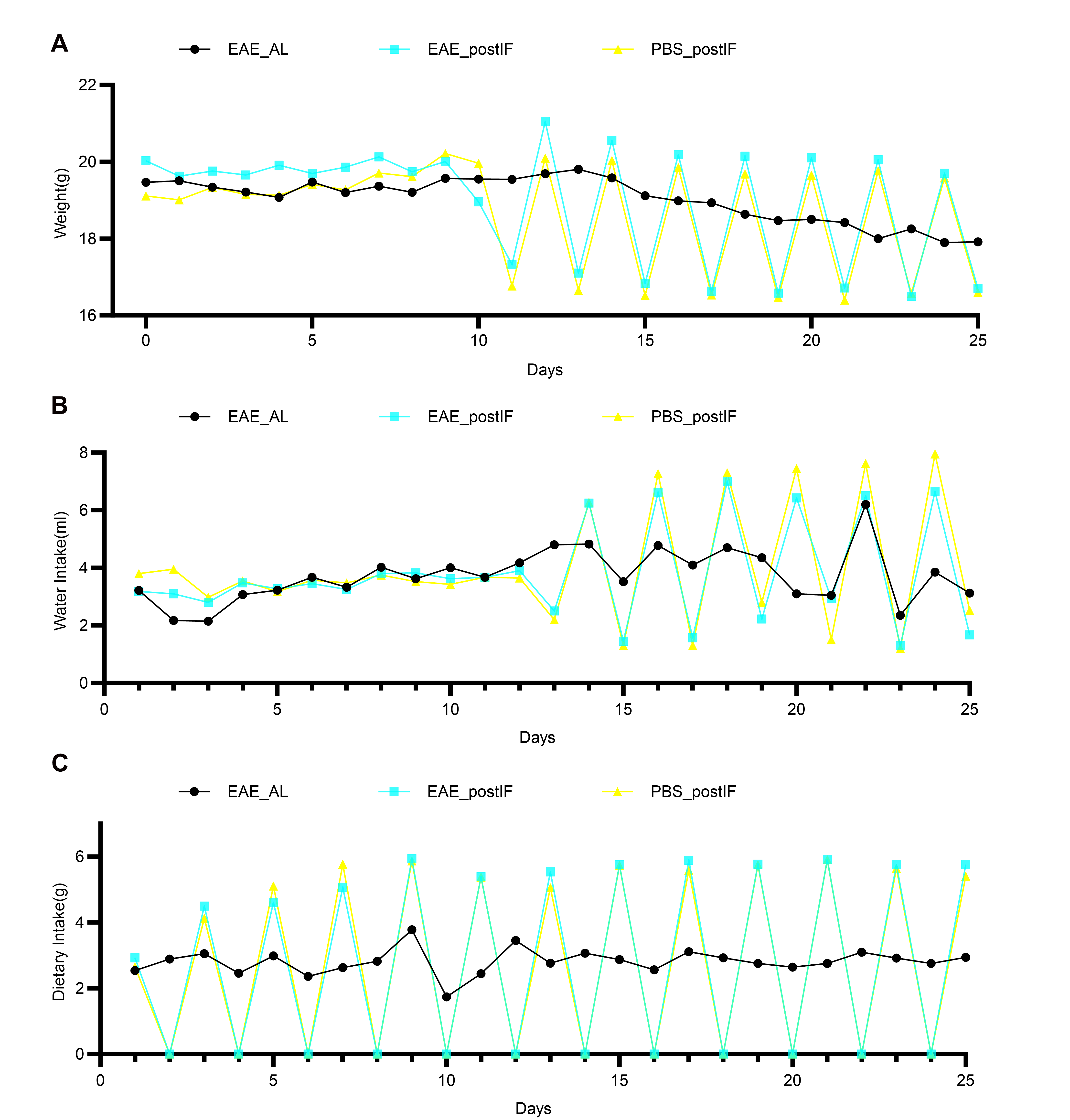
**

**Figure S4. Body weight, water, and food intake in EAE_postIF and PBS_postIF mice. Related to Figure 4. (A–C)** Time-course curves of body weight **(A)**, water intake **(B),** and food intake **(C)** for EAE_AL, EAE_postIF, and PBS_postIF groups (n=6 per group). No significant differences in overall intake were observed between EAE_postIF and PBS_postIF groups, indicating good tolerability of IF in both healthy and diseased mice. Data are presented as mean ± SEM.


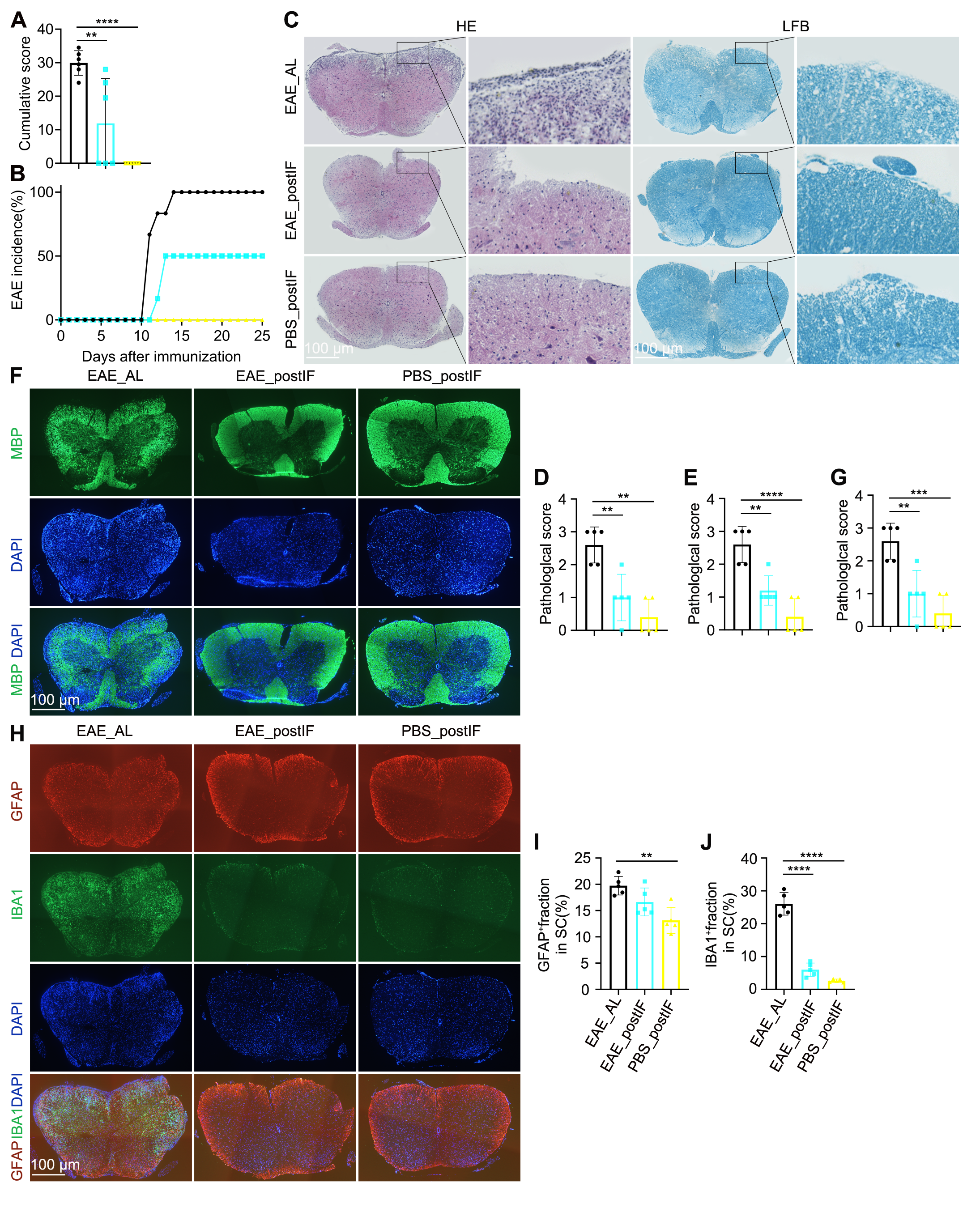
**Figure S5. Spinal cord pathology after IF in EAE and PBS_postIF groups. Related to Figure 4. (A)** Cumulative clinical scores of EAE_AL, EAE_postIF, and PBS_postIF mice. Data represent mean ± SEM and were analyzed using one-way ANOVA with Tukey’s post hoc test. **(B)** EAE incidence over time during disease progression in the same groups **(C)** Representative HE and LFB staining of lumbar spinal cords from EAE_AL, EAE_postIF, and PBS_postIF groups. (**D–E**) Quantification of inflammation (H&E) and demyelination (LFB) scores. **(F)** MBP and DAPI immunofluorescence. **(G)** Quantification of inflammation (MBP). **(H)** GFAP (red), IBA1 (green), and DAPI (blue) immunofluorescence. **(I–J)** Quantification of GFAP⁺ and IBA1⁺ areas. Post-onset IF significantly reduced neuroinflammation and demyelination in EAE_postIF mice, while PBS_postIF mice remained largely unaffected. Data are presented as mean ± SEM; *P<0.05, **P<0.01, ***P<0.001. scale bar = 100 μm.


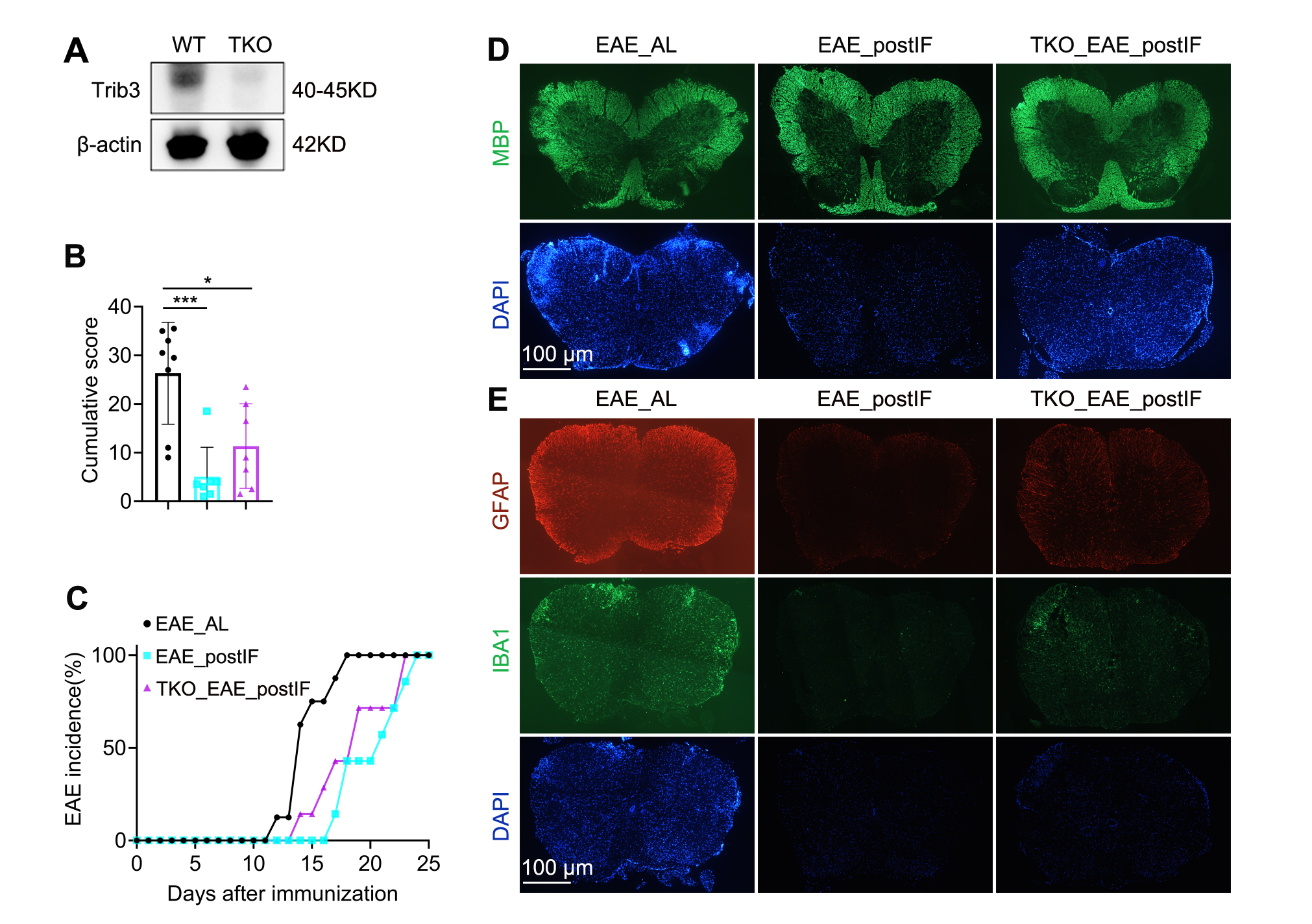


**Figure S6. Spinal cord demyelination and glial activation in TKO_EAE_postIF mice. Related to Figure 5. (A)** Western blot validation showing loss of TRIB3 protein in homozygous knockout mice. **(B)** Cumulative clinical scores of EAE_AL, EAE_postIF, and TKO_EAE_postIF mice. Data represent mean ± SEM and were analyzed using one-way ANOVA followed by Tukey’s post hoc test (*p* < 0.05). **(C)** EAE incidence over time during disease progression in the same groups. **(D)** Representative MBP and DAPI staining in EAE_AL, EAE_postIF, and TKO_EAE_postIF groups. **(E)** GFAP (red), IBA1 (green), and DAPI (blue) staining in the same groups. Loss of TRIB3 impaired the protective effect of IF on demyelination and glial activation. scale bar = 100 μm.


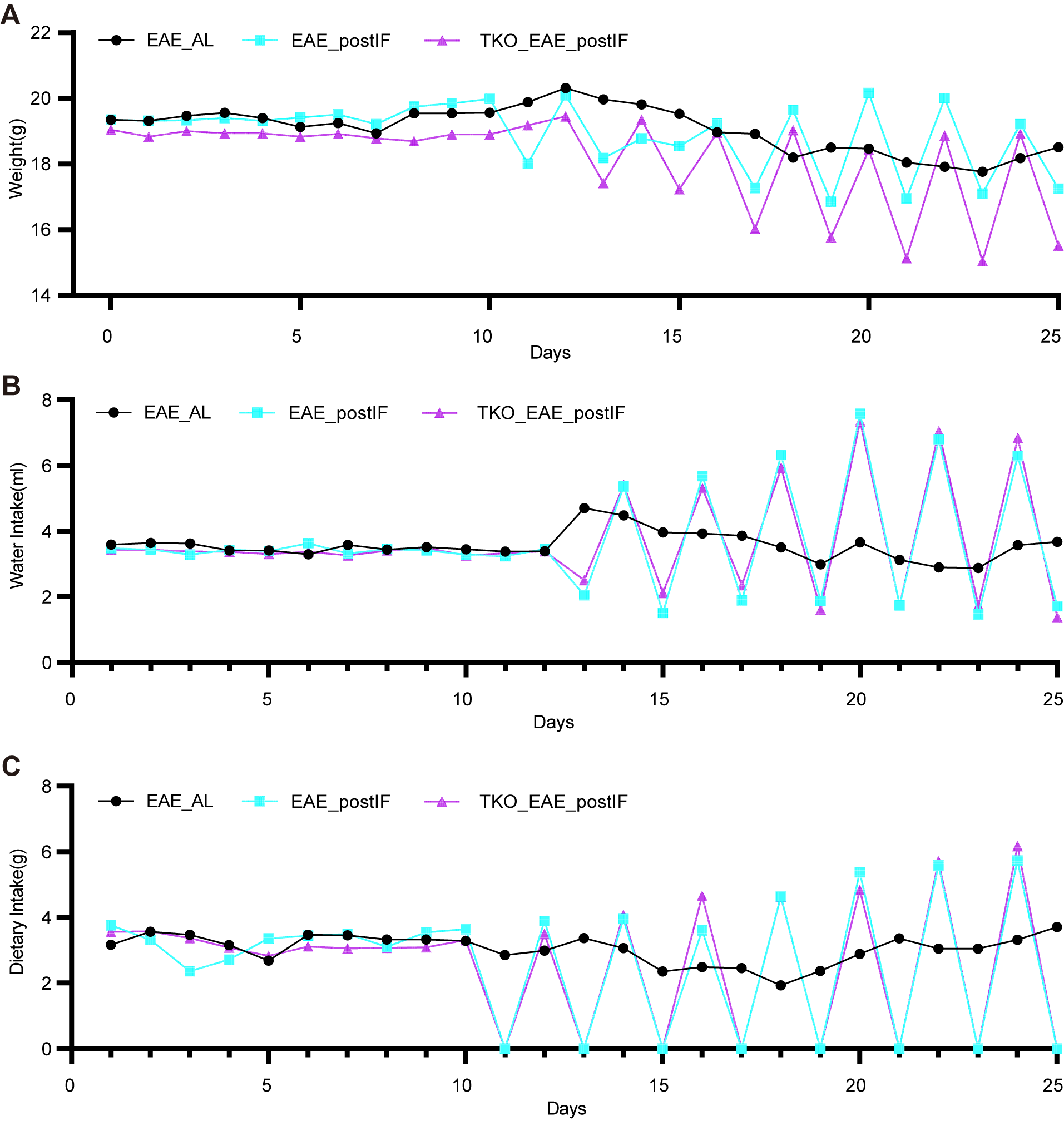
**Figure S7. Body weight, water, and food intake in TKO_EAE_postIF mice.** **Related to Figure 5.** (**A–C)** Time-course curves of body weight **(A),** water intake (**B**), and food intake (**C**) for EAE_AL, EAE_postIF, and TKO_EAE_postIF groups (n=8–10 per group). TRIB3 knockout (TKO) mice tolerated IF similarly to wild-type controls, with comparable intake and weight trends. Data are presented as mean ± SEM.


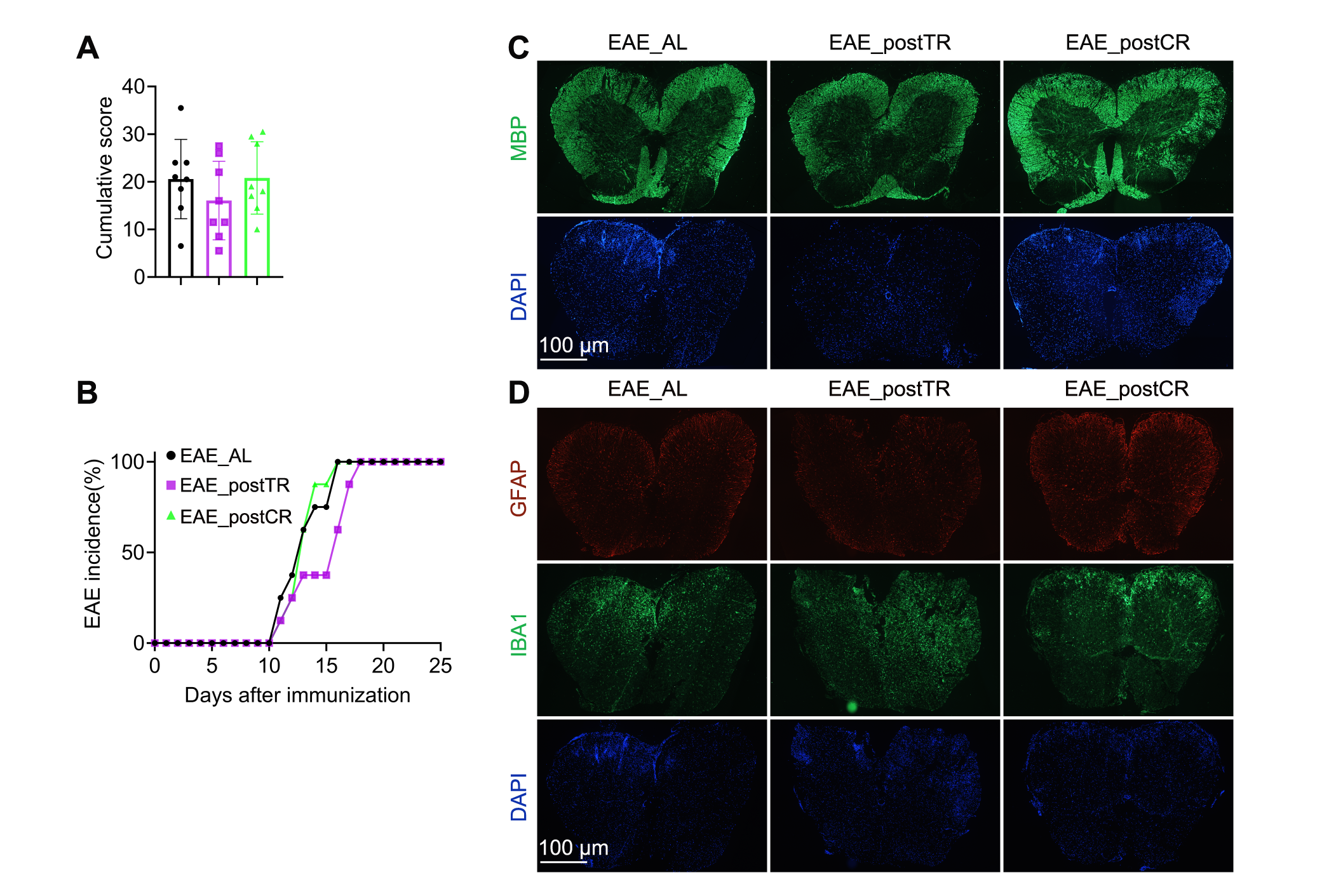


**Figure S8. Spinal cord demyelination and glial activation after TR and CR interventions. Related to Figure 7.** **(A)** Cumulative clinical scores of mice in EAE_AL, EAE_postTR, and EAE_postCR groups. Data represent mean ± SEM. Statistical analysis was performed by one-way ANOVA followed by Tukey’s post hoc test. **(B)** EAE incidence over time during disease progression in the same groups. **(C)** MBP and DAPI immunofluorescence in lumbar spinal cords from EAE_AL, EAE_postTR, and EAE_postCR groups. **(D)** GFAP (red), IBA1 (green), and DAPI (blue) staining in the same groups. TR and CR interventions showed minimal impact on demyelination and glial activation compared to EAE_AL. scale bar = 100 μm.


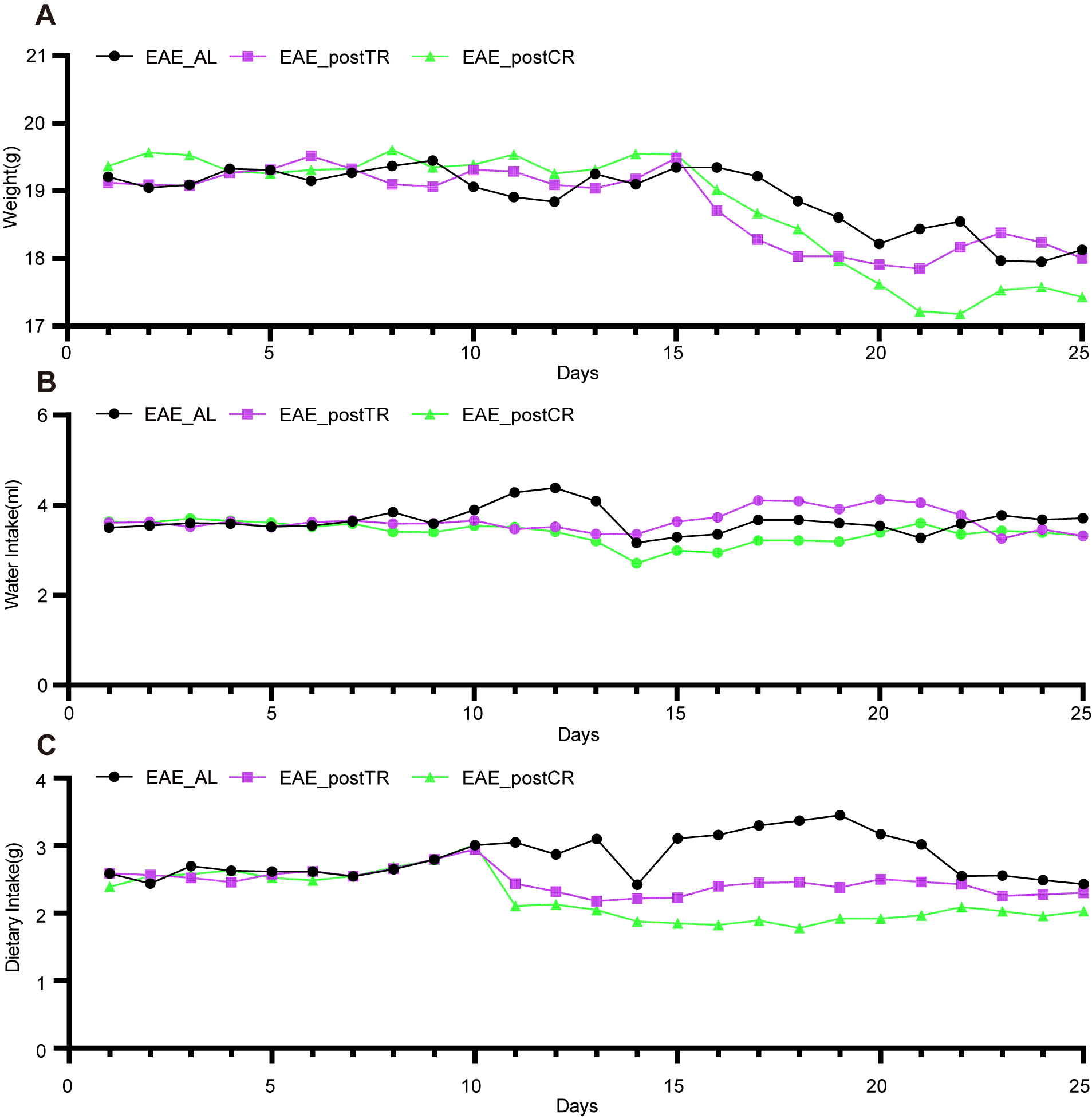


**Figure S9. Body weight, water, and food intake under TR and CR interventions. Related to Figure 7.** (**A–C**) Time-course curves of body weight (**A**), water intake (**B**), and food intake (**C**) for EAE_AL, EAE_postTR, and EAE_postCR groups (n=6 per group). Both TR and CR regimens were well-tolerated, with stable intake profiles. Data are presented as mean ± SEM.

| Antibodies | Source | Identifier |
| --- | --- | --- |
| IBA1, rabbit pAb | Abcam | Cat# ab5076; RRID: AB_2224402 |
| GFAP, mouse mAb | Abcam | Cat# ab7260; RRID: AB_305808 |
| MBP, rabbit pAb | Abcam | Cat# ab40390; RRID: AB_94778 |

**Table S1.** **Primary antibodies for immunofluorescence used in this study.**

| Antibodies | Source | Identifier |
| --- | --- | --- |
| CD4 (GK1.5), mouse mAb, PerCP-Cy5.5 | BioLegend | Cat# 100540; RRID: AB_312764 |
| IL-17A (TC11-18H10.1), rat mAb, APC | BioLegend | Cat# 506907; RRID: AB_315306 |
| IFN-γ (XMG1.2), rat mAb, PE | BioLegend | Cat# 505807; RRID: AB_315204 |
| FoxP3 (FJK-16s), rat mAb, PE | Thermo Fisher (eBioscience) | Cat# 25-5773-82; RRID: AB_10804767 |

**Table S2. Antibodies used for flow cytometry.**

**Table S3. Primary antibodies for western blotting used in this study.**

| Antibodies | Source | Identifier |
| --- | --- | --- |
| TRIB3, mouse mAb | Abnova | Cat# H00057761-M03 |
| p-eIF2α (Ser51), rabbit mAb | Beyotime | Cat# AF1237; RRID: AB_3094918 |
| ATF4, rabbit pAb | Proteintech | Cat# 10835-1-AP; RRID: AB_2058600 |
| CHOP, rabbit mAb | HUABIO | Cat# ET1703-05; RRID: AB_3070363 |
| p-AKT (Ser473), mouse mAb | Proteintech | Cat# 66444-1-Ig; RRID: AB_2782958 |
| AKT (pan), mouse mAb | Affinity | Cat# AF6261; RRID: AB_2835121 |
| PI3K p85α, mouse mAb | Affinity | Cat# AF6241; RRID: AB_2835340 |
| p-PI3K p85α (Tyr607), mouse mAb | Affinity | Cat# AF3241; RRID: AB_2834667 |
| p62/SQSTM1, rabbit mAb | Cell Signaling Tech. | Cat# 23214; RRID: AB_2798858 |
| mTOR (total), rabbit mAb | Abcam | Cat# ab109268; RRID: AB_10888105 |
| p-mTOR (Ser2448), rabbit mAb | Abcam | Cat# ab32028; RRID: AB_881283 |
| β-actin, mouse mAb | Abcam | Cat# ab8226; RRID: AB_2305186 |

**Table S4 Primers for Trib3 genotyping**

| Primer name | Primer sequence |
| --- | --- |
| F1 | 5′-ACTGGTCCTGTGTTTGGTCTCCAAG-3′ |
| R1 | 5′-CCTGTCAGATACCTTGGACTTGGAC-3′ |
| F2 | 5′-ATCCAGGGGAGCAGAGTAACTTAG-3′ |
