# Supplementary figures and images for: Post-onset intermittent fasting attenuates neuroinflammation and demyelination via a TRIB3–PERK–autophagy axis in an EAE model of multiple sclerosis
Source: J Neuroinflammation. 2025 Nov 27;22:301. doi: 10.1186/s12974-025-03640-y (PMC12752437; doi:10.1186/s12974-025-03640-y)

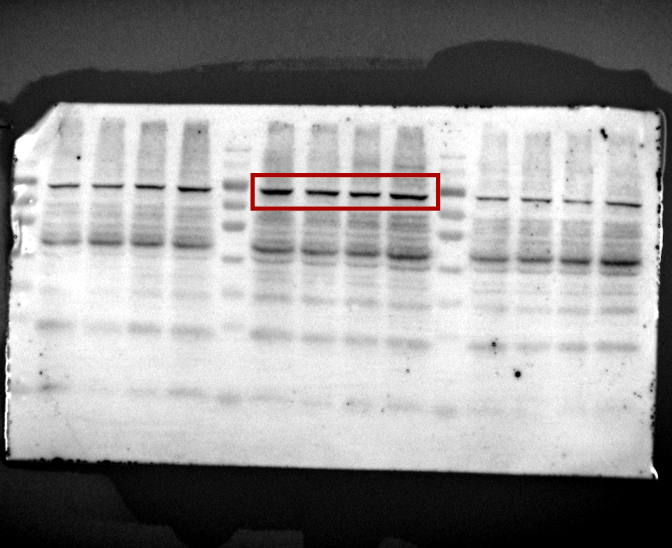

Supplement: Supplementary file 2 — Supplementary Material 2. Supplementary Material 2 includes original data supporting the main figures, including full uncropped gels and blots, as well as other raw experimental data. [file 12974_2025_3640_MOESM2_ESM.zip › Supplementary Material 2/Data for Figure 3/AKT.tif]

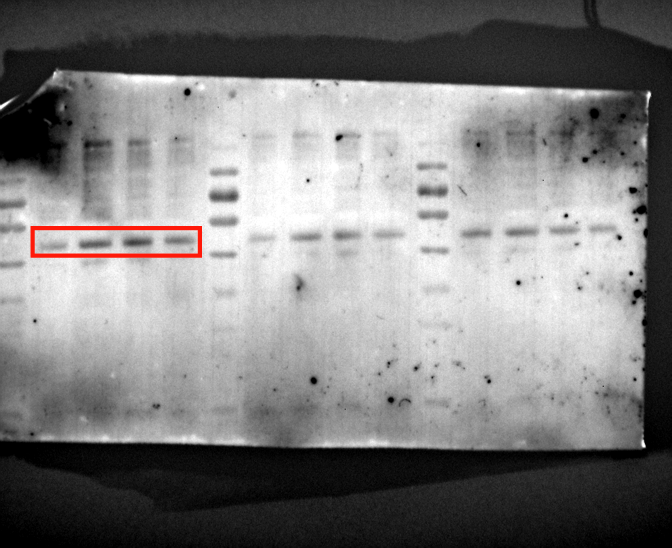

Supplement: Supplementary file 2 — Supplementary Material 2. Supplementary Material 2 includes original data supporting the main figures, including full uncropped gels and blots, as well as other raw experimental data. [file 12974_2025_3640_MOESM2_ESM.zip › Supplementary Material 2/Data for Figure 3/ATF4.tif]

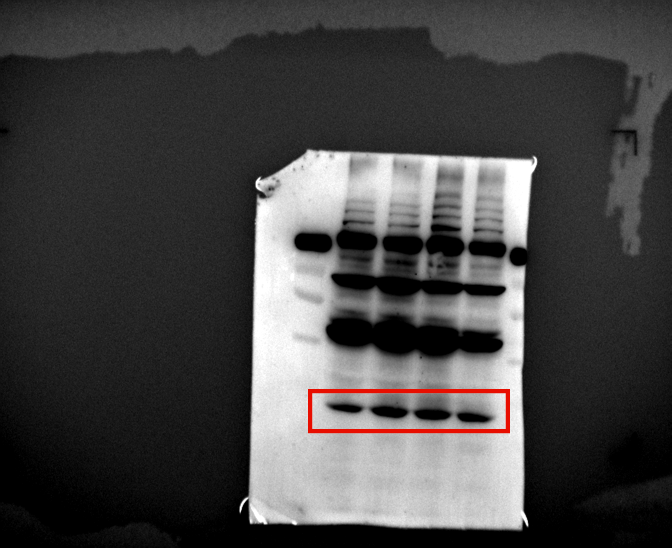

Supplement: Supplementary file 2 — Supplementary Material 2. Supplementary Material 2 includes original data supporting the main figures, including full uncropped gels and blots, as well as other raw experimental data. [file 12974_2025_3640_MOESM2_ESM.zip › Supplementary Material 2/Data for Figure 3/CHOP.tif]

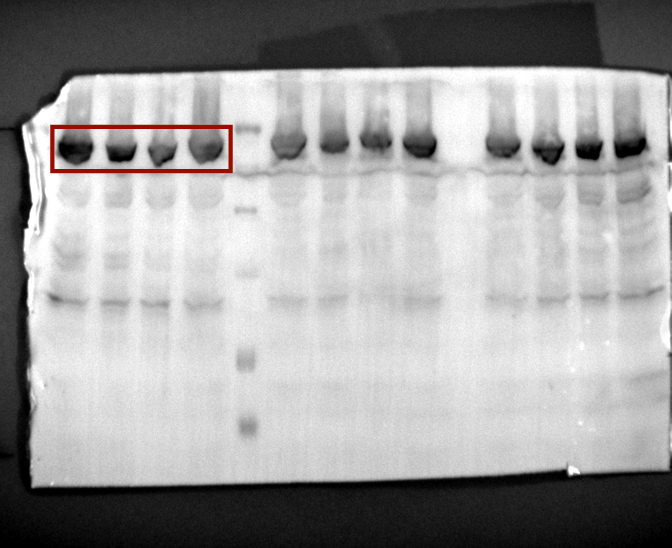

Supplement: Supplementary file 2 — Supplementary Material 2. Supplementary Material 2 includes original data supporting the main figures, including full uncropped gels and blots, as well as other raw experimental data. [file 12974_2025_3640_MOESM2_ESM.zip › Supplementary Material 2/Data for Figure 3/mTOR.tif]

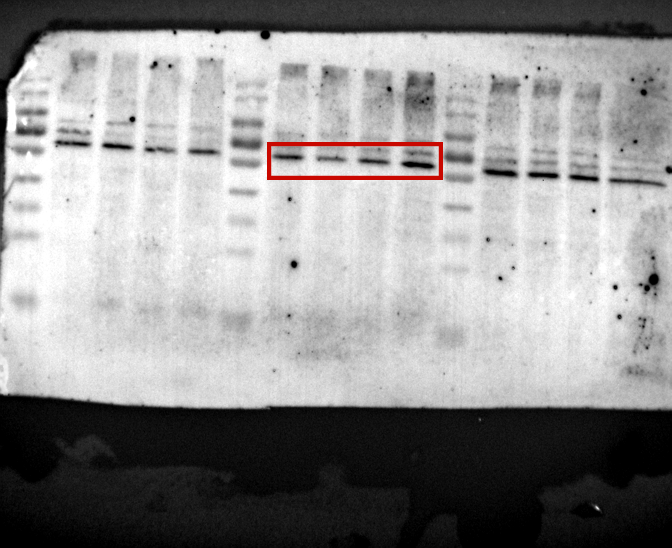

Supplement: Supplementary file 2 — Supplementary Material 2. Supplementary Material 2 includes original data supporting the main figures, including full uncropped gels and blots, as well as other raw experimental data. [file 12974_2025_3640_MOESM2_ESM.zip › Supplementary Material 2/Data for Figure 3/p-AKT.tif]

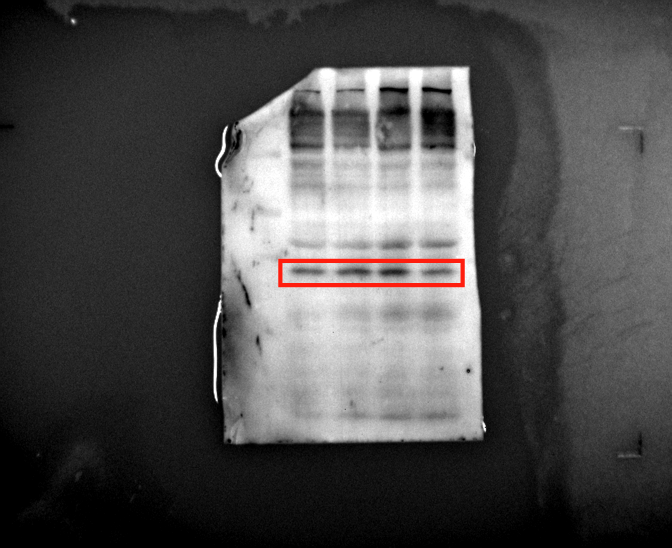

Supplement: Supplementary file 2 — Supplementary Material 2. Supplementary Material 2 includes original data supporting the main figures, including full uncropped gels and blots, as well as other raw experimental data. [file 12974_2025_3640_MOESM2_ESM.zip › Supplementary Material 2/Data for Figure 3/p-eIF2α.tif]

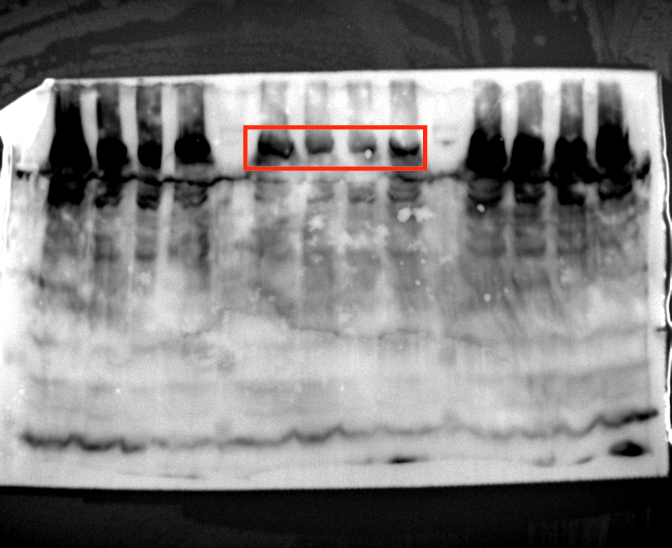

Supplement: Supplementary file 2 — Supplementary Material 2. Supplementary Material 2 includes original data supporting the main figures, including full uncropped gels and blots, as well as other raw experimental data. [file 12974_2025_3640_MOESM2_ESM.zip › Supplementary Material 2/Data for Figure 3/p-mTOR.tif]

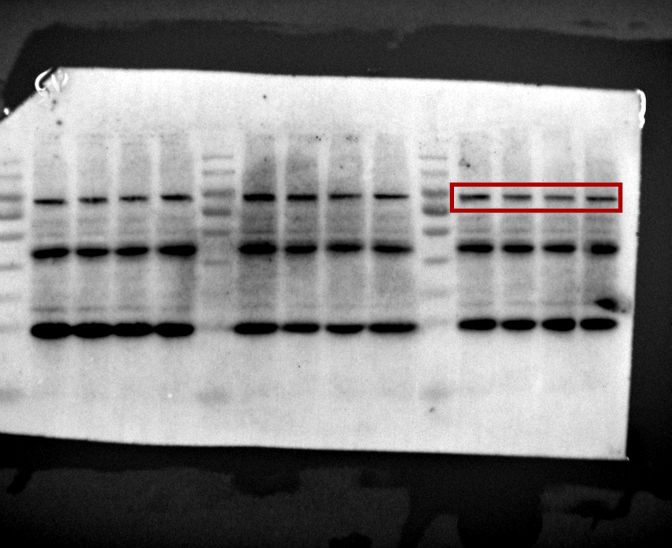

Supplement: Supplementary file 2 — Supplementary Material 2. Supplementary Material 2 includes original data supporting the main figures, including full uncropped gels and blots, as well as other raw experimental data. [file 12974_2025_3640_MOESM2_ESM.zip › Supplementary Material 2/Data for Figure 3/p-PI3K.tif]

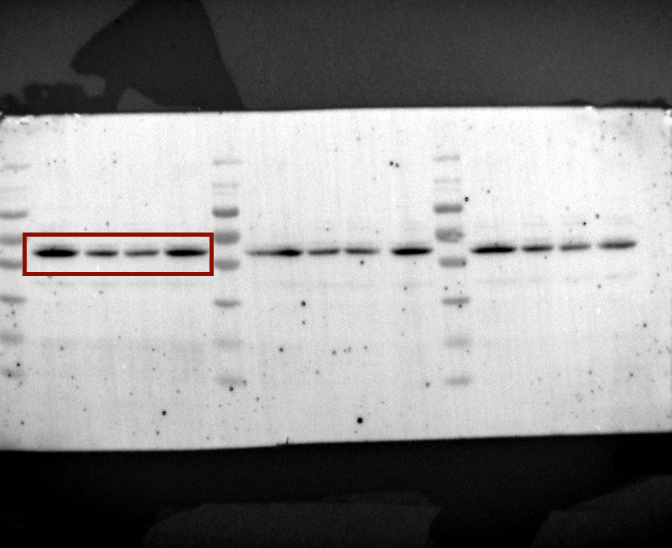

Supplement: Supplementary file 2 — Supplementary Material 2. Supplementary Material 2 includes original data supporting the main figures, including full uncropped gels and blots, as well as other raw experimental data. [file 12974_2025_3640_MOESM2_ESM.zip › Supplementary Material 2/Data for Figure 3/p62.tif]

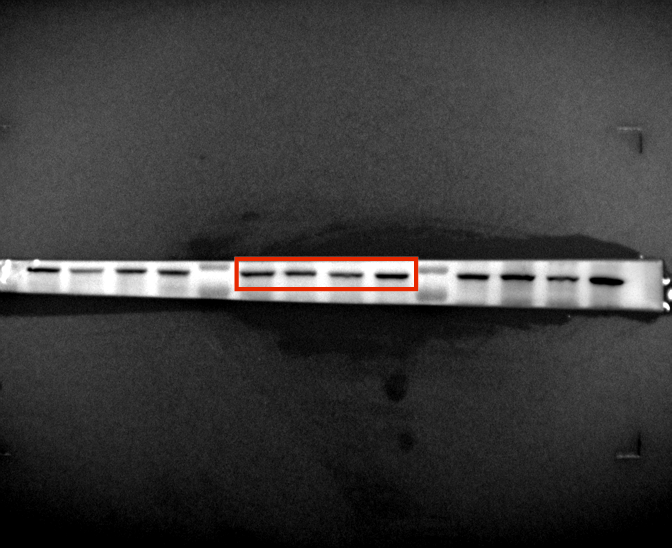

Supplement: Supplementary file 2 — Supplementary Material 2. Supplementary Material 2 includes original data supporting the main figures, including full uncropped gels and blots, as well as other raw experimental data. [file 12974_2025_3640_MOESM2_ESM.zip › Supplementary Material 2/Data for Figure 3/PI3K.tif]

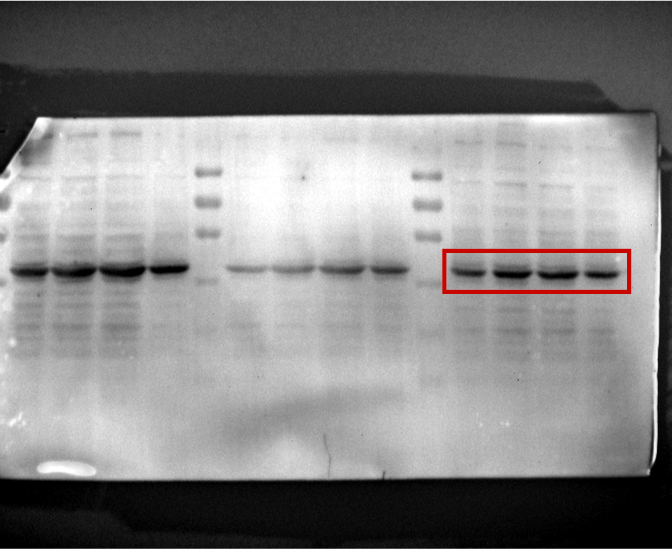

Supplement: Supplementary file 2 — Supplementary Material 2. Supplementary Material 2 includes original data supporting the main figures, including full uncropped gels and blots, as well as other raw experimental data. [file 12974_2025_3640_MOESM2_ESM.zip › Supplementary Material 2/Data for Figure 3/TRIB3.tif]

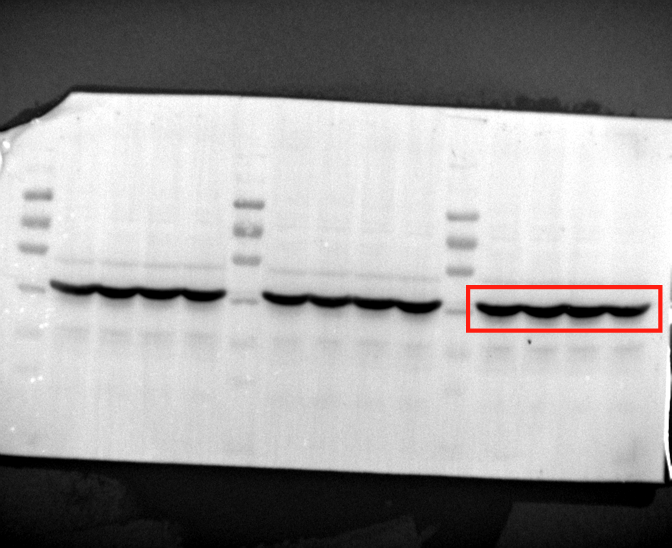

Supplement: Supplementary file 2 — Supplementary Material 2. Supplementary Material 2 includes original data supporting the main figures, including full uncropped gels and blots, as well as other raw experimental data. [file 12974_2025_3640_MOESM2_ESM.zip › Supplementary Material 2/Data for Figure 3/β-actin.tif]

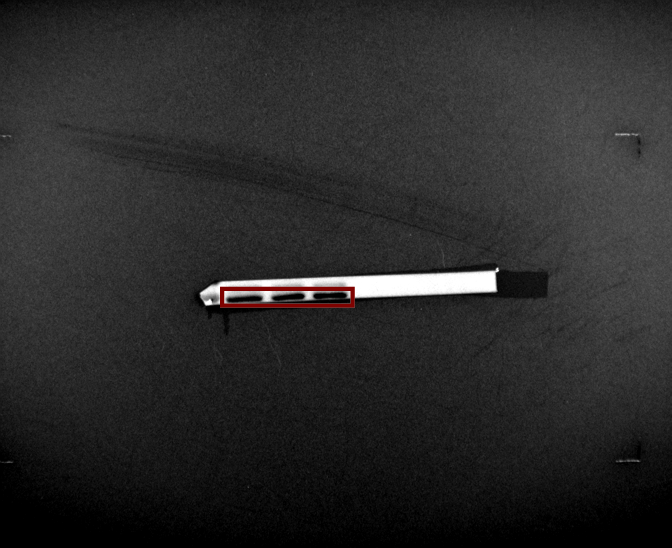

Supplement: Supplementary file 2 — Supplementary Material 2. Supplementary Material 2 includes original data supporting the main figures, including full uncropped gels and blots, as well as other raw experimental data. [file 12974_2025_3640_MOESM2_ESM.zip › Supplementary Material 2/Data for Figure 4 C-D/AKT.tif]

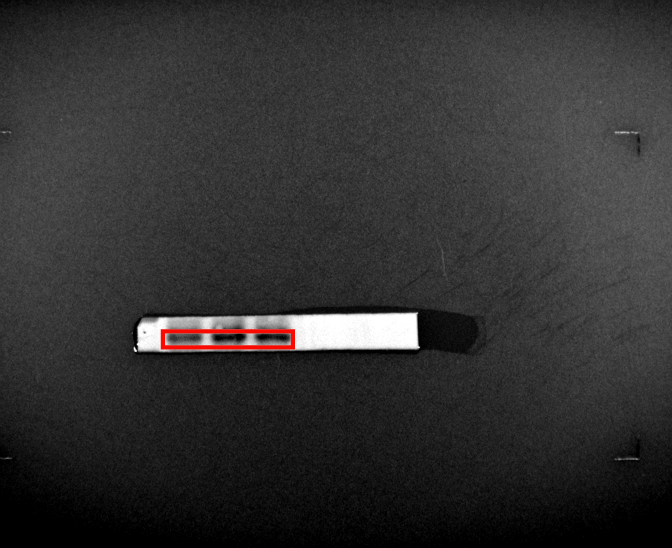

Supplement: Supplementary file 2 — Supplementary Material 2. Supplementary Material 2 includes original data supporting the main figures, including full uncropped gels and blots, as well as other raw experimental data. [file 12974_2025_3640_MOESM2_ESM.zip › Supplementary Material 2/Data for Figure 4 C-D/ATF4.tif]

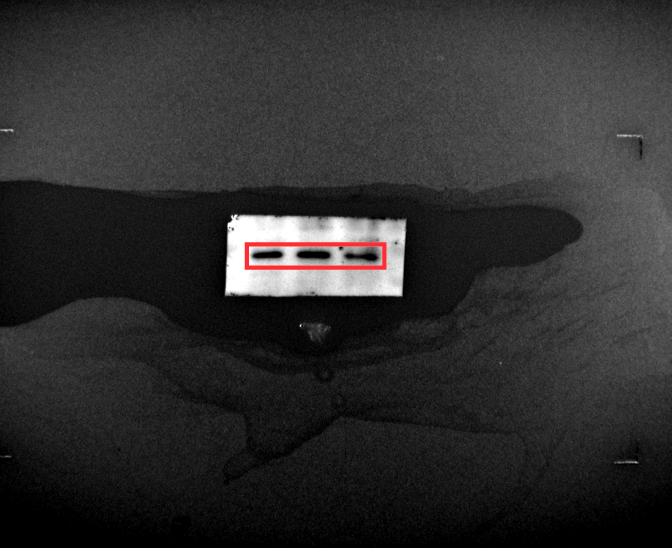

Supplement: Supplementary file 2 — Supplementary Material 2. Supplementary Material 2 includes original data supporting the main figures, including full uncropped gels and blots, as well as other raw experimental data. [file 12974_2025_3640_MOESM2_ESM.zip › Supplementary Material 2/Data for Figure 4 C-D/CHOP.tif]

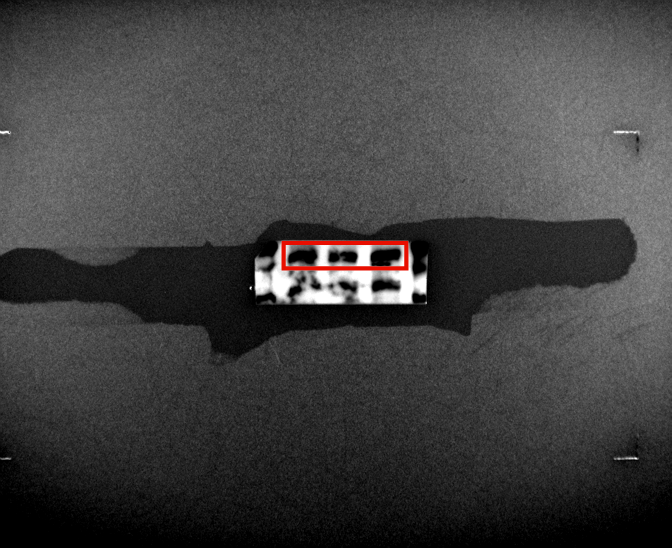

Supplement: Supplementary file 2 — Supplementary Material 2. Supplementary Material 2 includes original data supporting the main figures, including full uncropped gels and blots, as well as other raw experimental data. [file 12974_2025_3640_MOESM2_ESM.zip › Supplementary Material 2/Data for Figure 4 C-D/mTOR.tif]

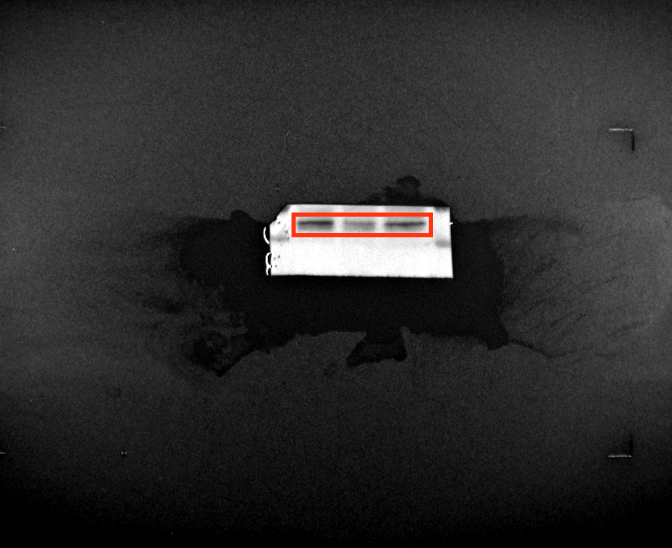

Supplement: Supplementary file 2 — Supplementary Material 2. Supplementary Material 2 includes original data supporting the main figures, including full uncropped gels and blots, as well as other raw experimental data. [file 12974_2025_3640_MOESM2_ESM.zip › Supplementary Material 2/Data for Figure 4 C-D/p-AKT.tif]

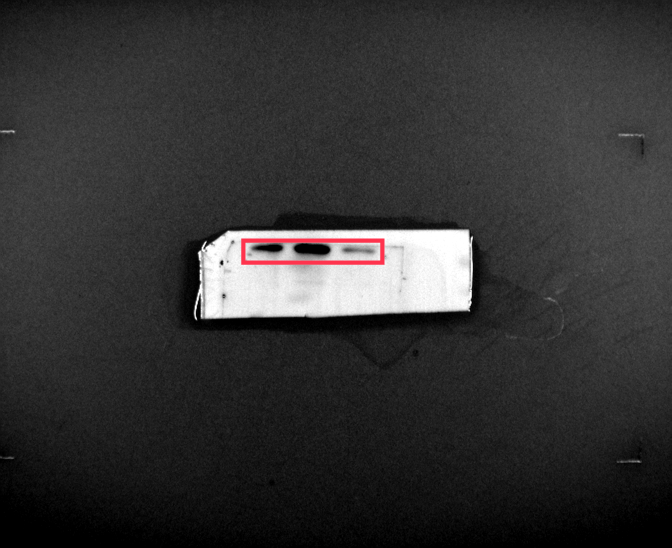

Supplement: Supplementary file 2 — Supplementary Material 2. Supplementary Material 2 includes original data supporting the main figures, including full uncropped gels and blots, as well as other raw experimental data. [file 12974_2025_3640_MOESM2_ESM.zip › Supplementary Material 2/Data for Figure 4 C-D/p-eIF2α.tif]

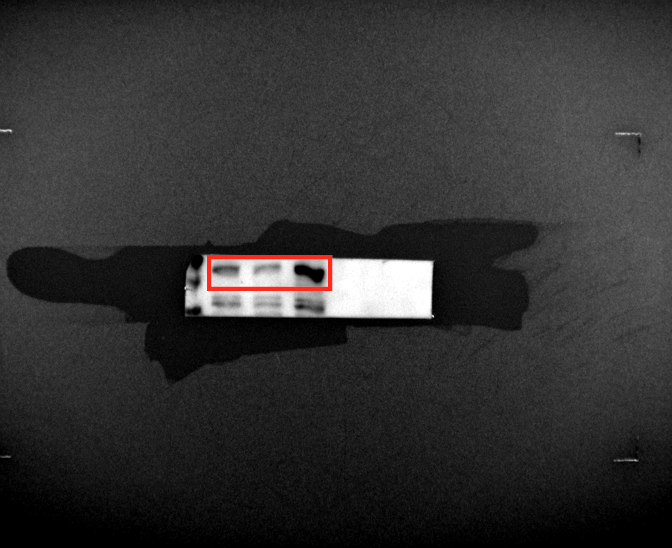

Supplement: Supplementary file 2 — Supplementary Material 2. Supplementary Material 2 includes original data supporting the main figures, including full uncropped gels and blots, as well as other raw experimental data. [file 12974_2025_3640_MOESM2_ESM.zip › Supplementary Material 2/Data for Figure 4 C-D/p-mTOR.tif]

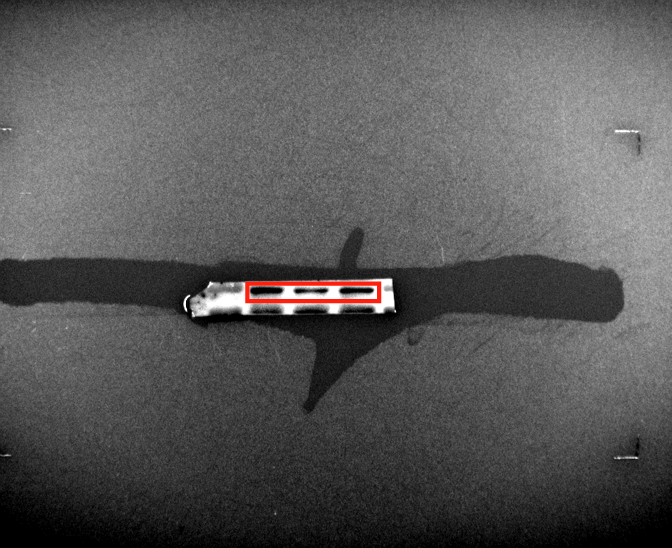

Supplement: Supplementary file 2 — Supplementary Material 2. Supplementary Material 2 includes original data supporting the main figures, including full uncropped gels and blots, as well as other raw experimental data. [file 12974_2025_3640_MOESM2_ESM.zip › Supplementary Material 2/Data for Figure 4 C-D/p-PI3K.tif]

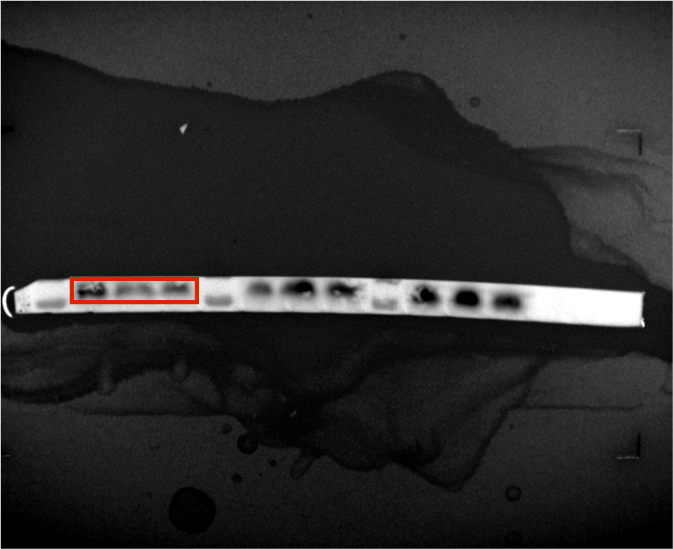

Supplement: Supplementary file 2 — Supplementary Material 2. Supplementary Material 2 includes original data supporting the main figures, including full uncropped gels and blots, as well as other raw experimental data. [file 12974_2025_3640_MOESM2_ESM.zip › Supplementary Material 2/Data for Figure 4 C-D/p62.tif]

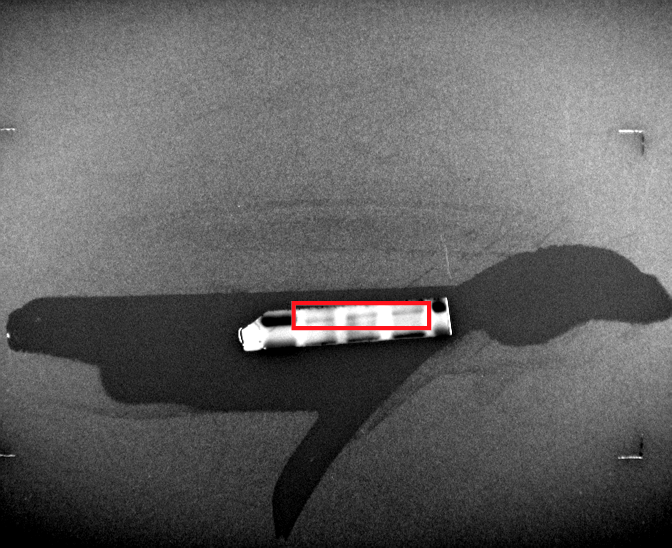

Supplement: Supplementary file 2 — Supplementary Material 2. Supplementary Material 2 includes original data supporting the main figures, including full uncropped gels and blots, as well as other raw experimental data. [file 12974_2025_3640_MOESM2_ESM.zip › Supplementary Material 2/Data for Figure 4 C-D/PI3K.tif]

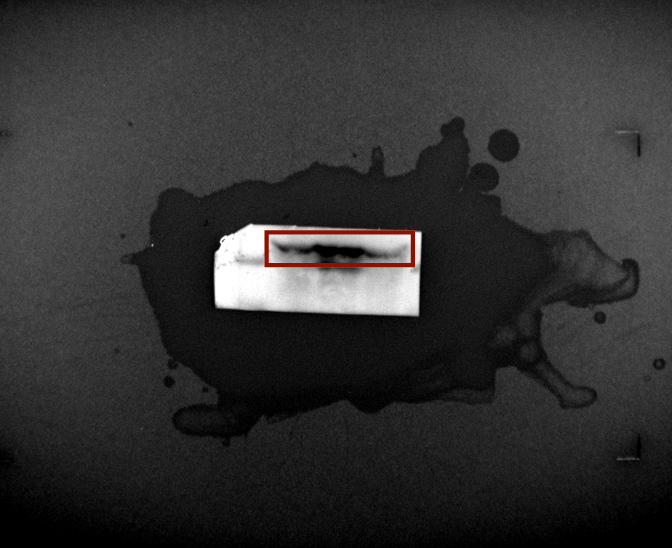

Supplement: Supplementary file 2 — Supplementary Material 2. Supplementary Material 2 includes original data supporting the main figures, including full uncropped gels and blots, as well as other raw experimental data. [file 12974_2025_3640_MOESM2_ESM.zip › Supplementary Material 2/Data for Figure 4 C-D/TRIB3.tif]

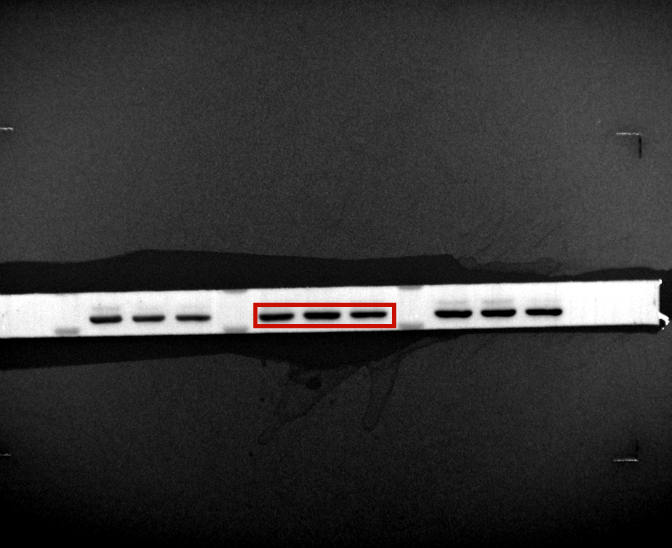

Supplement: Supplementary file 2 — Supplementary Material 2. Supplementary Material 2 includes original data supporting the main figures, including full uncropped gels and blots, as well as other raw experimental data. [file 12974_2025_3640_MOESM2_ESM.zip › Supplementary Material 2/Data for Figure 4 C-D/β-actin.tif]

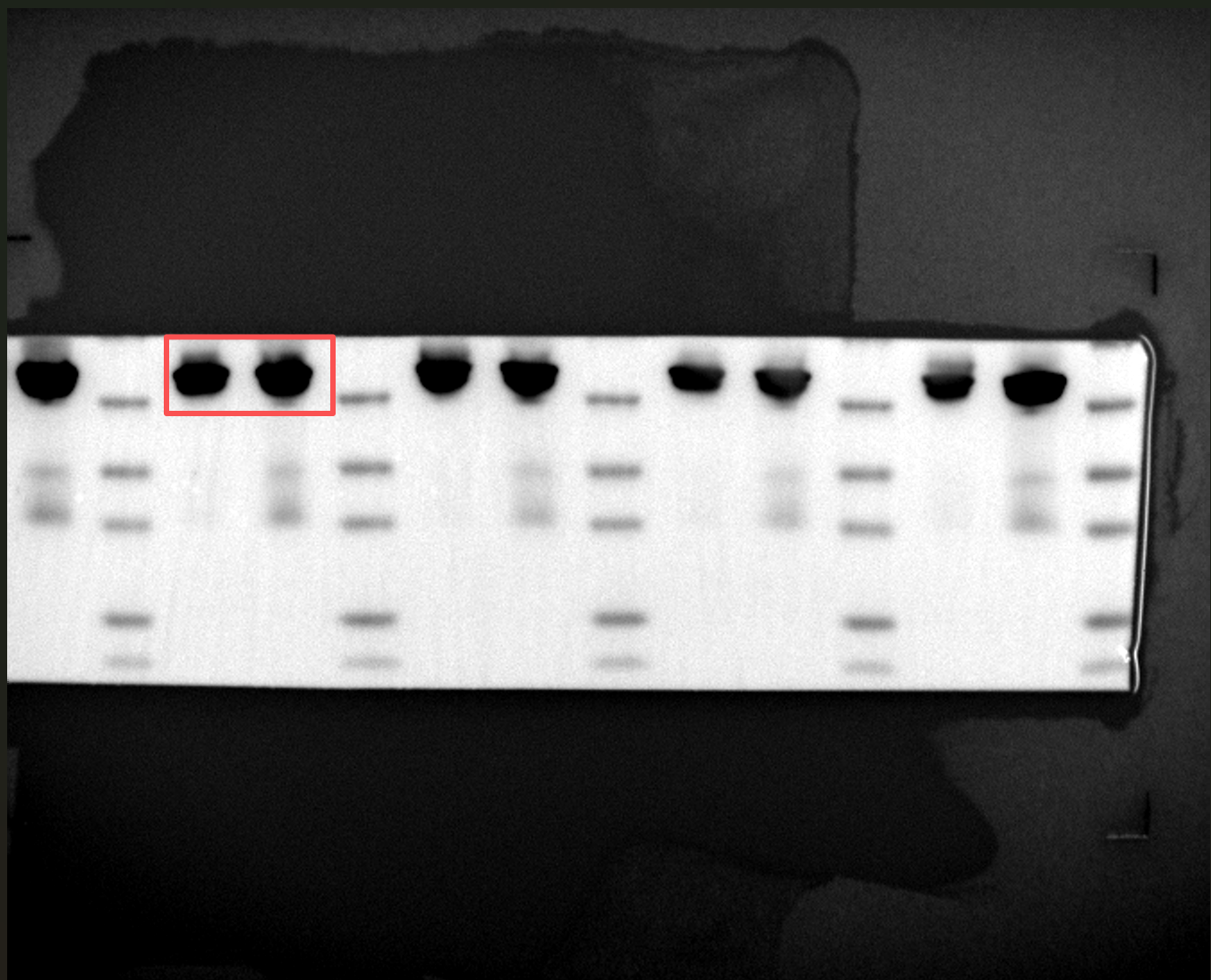

Supplement: Supplementary file 2 — Supplementary Material 2. Supplementary Material 2 includes original data supporting the main figures, including full uncropped gels and blots, as well as other raw experimental data. [file 12974_2025_3640_MOESM2_ESM.zip › Supplementary Material 2/Data for Figure 5–Figure Supplement 6 A/actin.png]

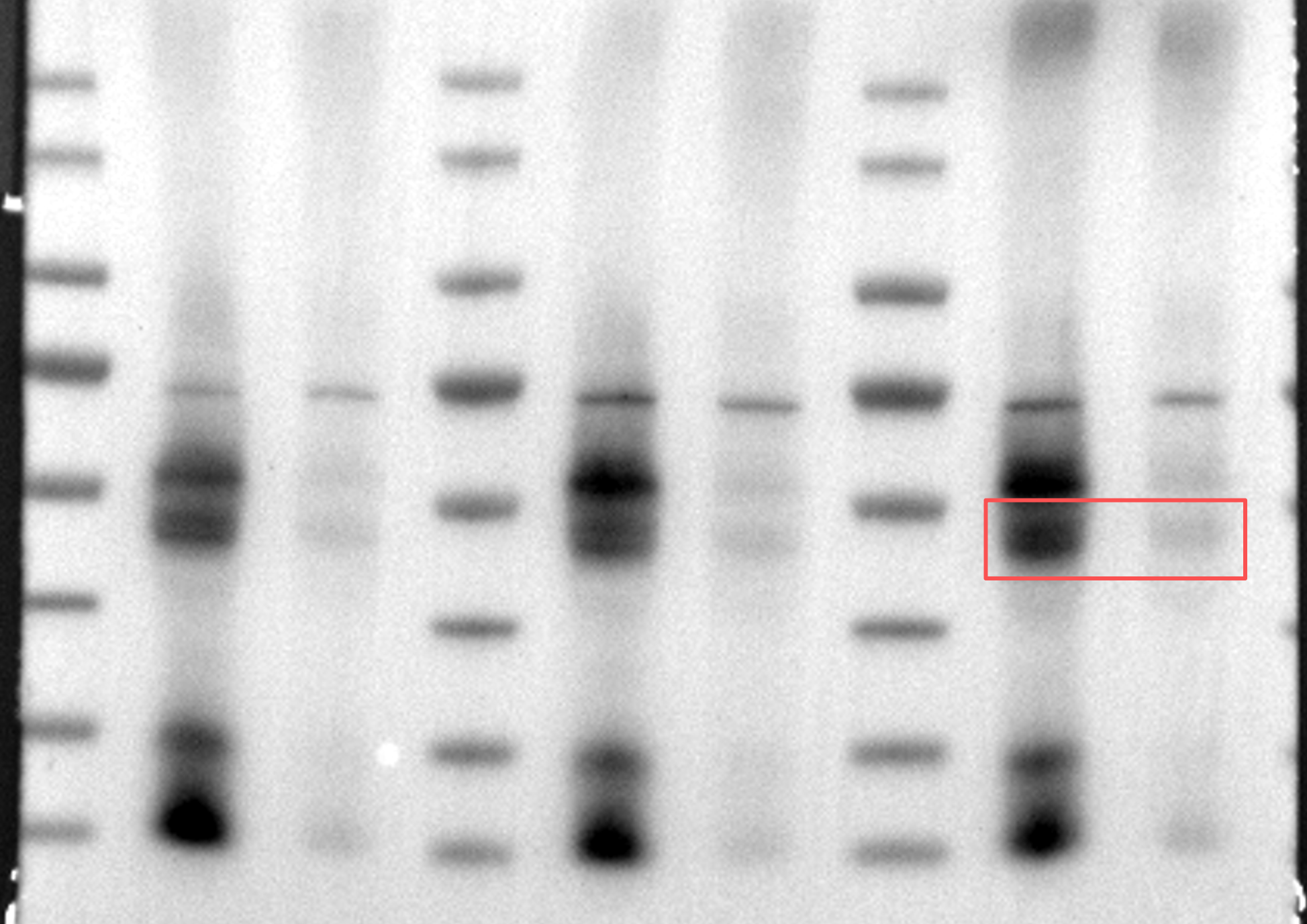

Supplement: Supplementary file 2 — Supplementary Material 2. Supplementary Material 2 includes original data supporting the main figures, including full uncropped gels and blots, as well as other raw experimental data. [file 12974_2025_3640_MOESM2_ESM.zip › Supplementary Material 2/Data for Figure 5–Figure Supplement 6 A/Trib3.png]

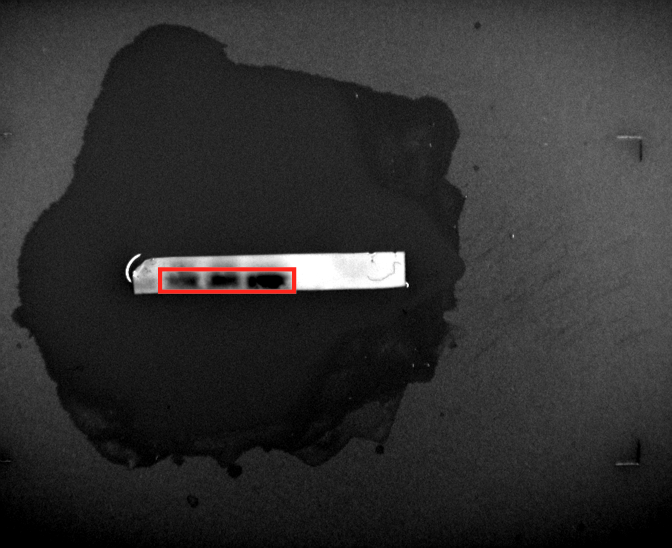

Supplement: Supplementary file 2 — Supplementary Material 2. Supplementary Material 2 includes original data supporting the main figures, including full uncropped gels and blots, as well as other raw experimental data. [file 12974_2025_3640_MOESM2_ESM.zip › Supplementary Material 2/Data for Figure 6/AKT.tif]

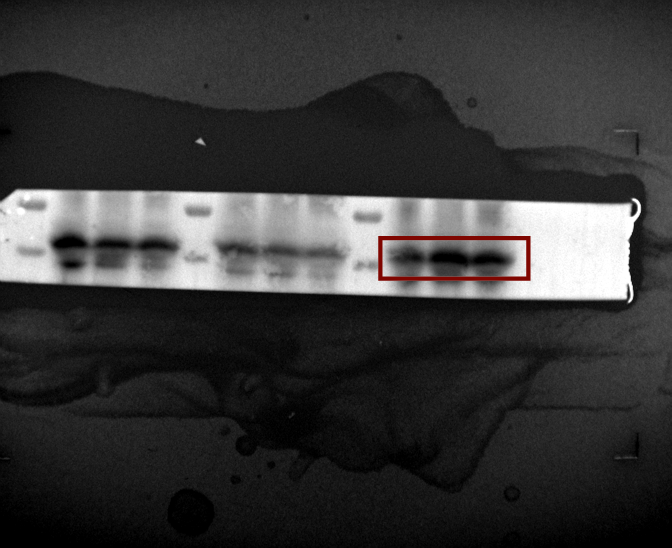

Supplement: Supplementary file 2 — Supplementary Material 2. Supplementary Material 2 includes original data supporting the main figures, including full uncropped gels and blots, as well as other raw experimental data. [file 12974_2025_3640_MOESM2_ESM.zip › Supplementary Material 2/Data for Figure 6/ATF4.tif]

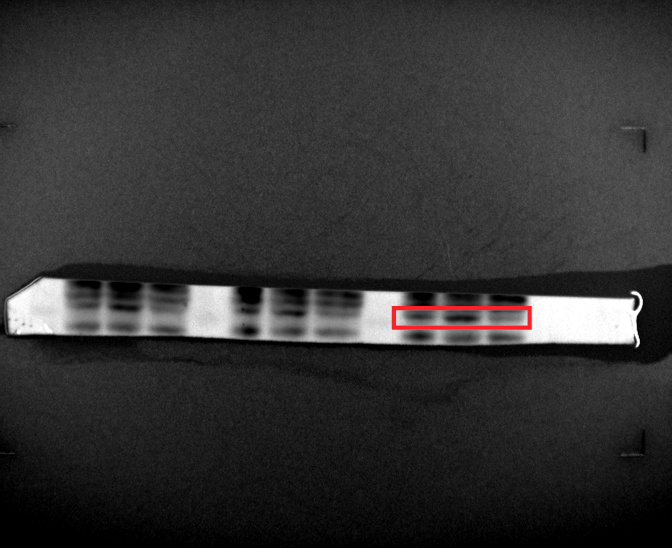

Supplement: Supplementary file 2 — Supplementary Material 2. Supplementary Material 2 includes original data supporting the main figures, including full uncropped gels and blots, as well as other raw experimental data. [file 12974_2025_3640_MOESM2_ESM.zip › Supplementary Material 2/Data for Figure 6/CHOP.tif]

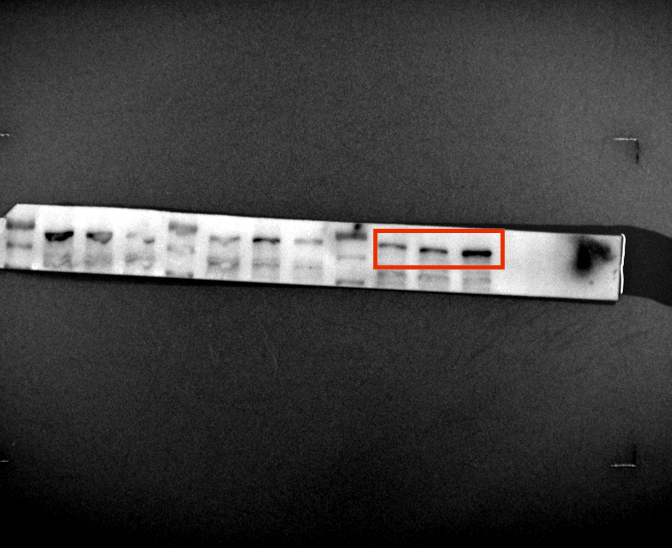

Supplement: Supplementary file 2 — Supplementary Material 2. Supplementary Material 2 includes original data supporting the main figures, including full uncropped gels and blots, as well as other raw experimental data. [file 12974_2025_3640_MOESM2_ESM.zip › Supplementary Material 2/Data for Figure 6/mTOR.tif]

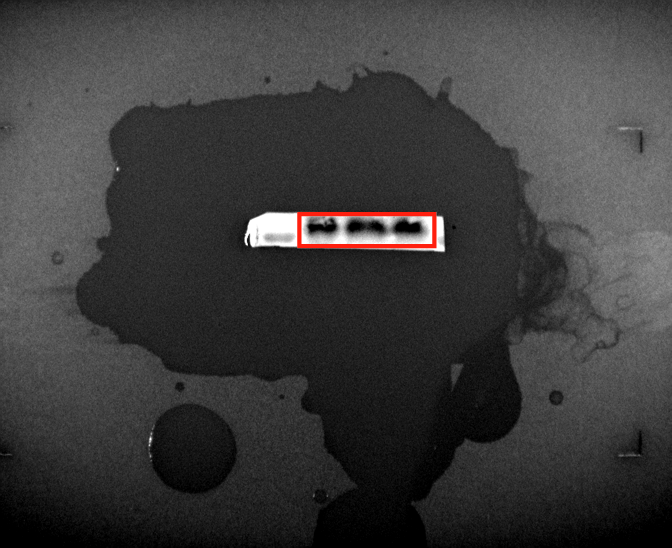

Supplement: Supplementary file 2 — Supplementary Material 2. Supplementary Material 2 includes original data supporting the main figures, including full uncropped gels and blots, as well as other raw experimental data. [file 12974_2025_3640_MOESM2_ESM.zip › Supplementary Material 2/Data for Figure 6/p-AKT.tif]

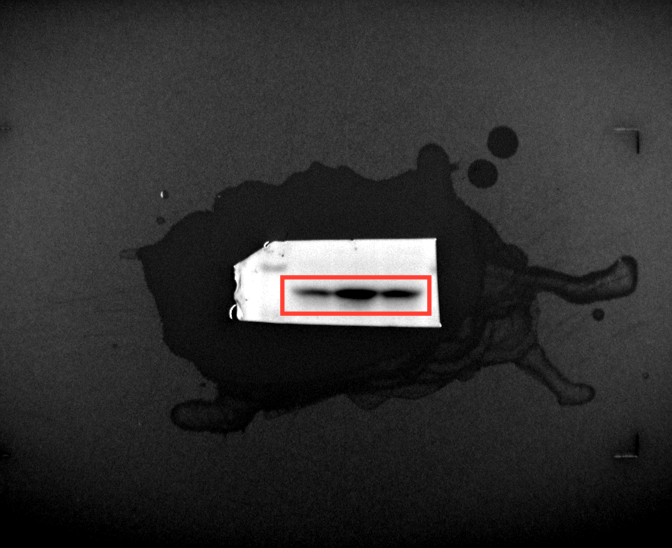

Supplement: Supplementary file 2 — Supplementary Material 2. Supplementary Material 2 includes original data supporting the main figures, including full uncropped gels and blots, as well as other raw experimental data. [file 12974_2025_3640_MOESM2_ESM.zip › Supplementary Material 2/Data for Figure 6/p-eIF2α.tif]

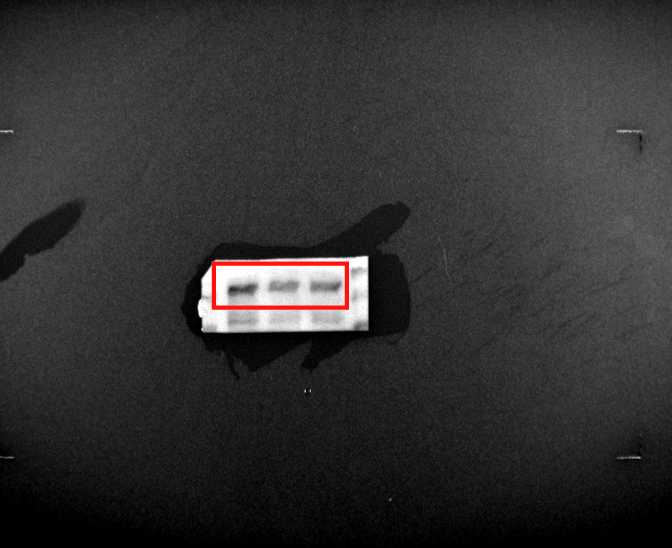

Supplement: Supplementary file 2 — Supplementary Material 2. Supplementary Material 2 includes original data supporting the main figures, including full uncropped gels and blots, as well as other raw experimental data. [file 12974_2025_3640_MOESM2_ESM.zip › Supplementary Material 2/Data for Figure 6/p-mTOR.tif]

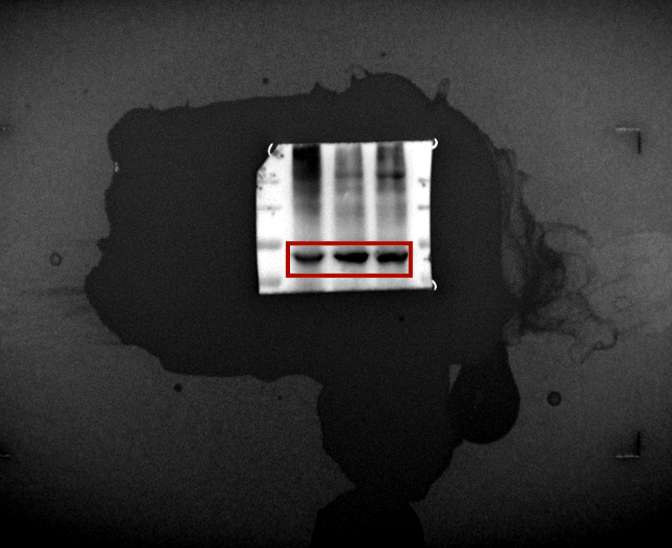

Supplement: Supplementary file 2 — Supplementary Material 2. Supplementary Material 2 includes original data supporting the main figures, including full uncropped gels and blots, as well as other raw experimental data. [file 12974_2025_3640_MOESM2_ESM.zip › Supplementary Material 2/Data for Figure 6/p-PI3K.tif]

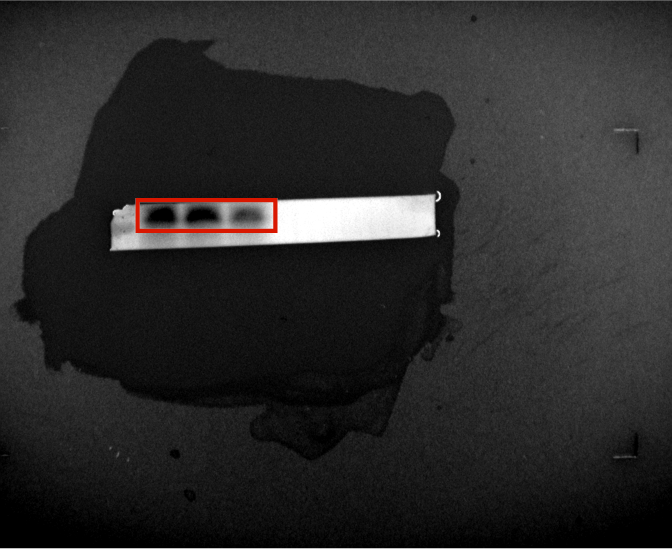

Supplement: Supplementary file 2 — Supplementary Material 2. Supplementary Material 2 includes original data supporting the main figures, including full uncropped gels and blots, as well as other raw experimental data. [file 12974_2025_3640_MOESM2_ESM.zip › Supplementary Material 2/Data for Figure 6/p62.tif]

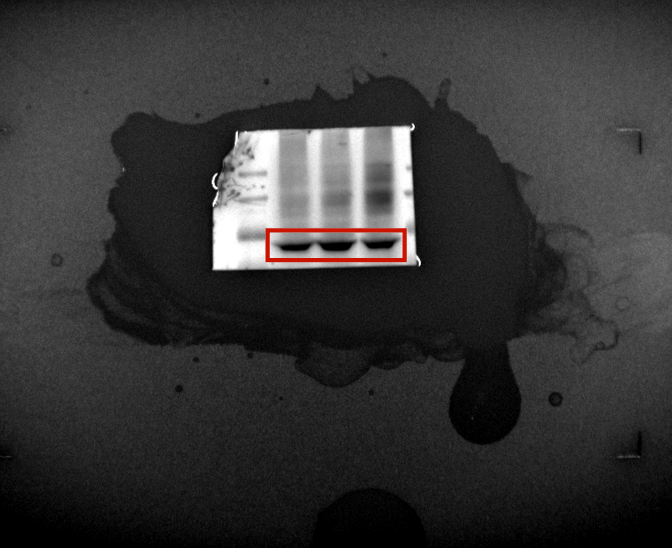

Supplement: Supplementary file 2 — Supplementary Material 2. Supplementary Material 2 includes original data supporting the main figures, including full uncropped gels and blots, as well as other raw experimental data. [file 12974_2025_3640_MOESM2_ESM.zip › Supplementary Material 2/Data for Figure 6/PI3K.tif]

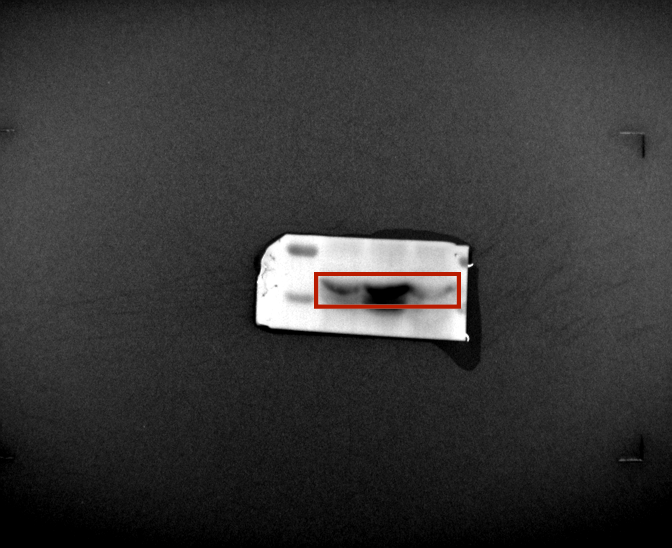

Supplement: Supplementary file 2 — Supplementary Material 2. Supplementary Material 2 includes original data supporting the main figures, including full uncropped gels and blots, as well as other raw experimental data. [file 12974_2025_3640_MOESM2_ESM.zip › Supplementary Material 2/Data for Figure 6/TRIB3.tif]

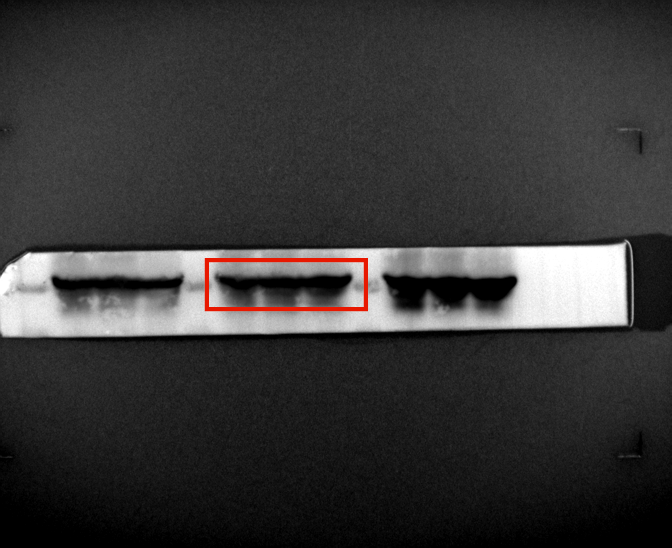

Supplement: Supplementary file 2 — Supplementary Material 2. Supplementary Material 2 includes original data supporting the main figures, including full uncropped gels and blots, as well as other raw experimental data. [file 12974_2025_3640_MOESM2_ESM.zip › Supplementary Material 2/Data for Figure 6/β-actin.tif]
